# Similarity in replication timing between polytene and diploid cells is associated with the organization of the *Drosophila* genome

**DOI:** 10.1371/journal.pone.0195207

**Published:** 2018-04-16

**Authors:** Tatyana D. Kolesnikova, Fedor P. Goncharov, Igor F. Zhimulev

**Affiliations:** 1 Institute of Molecular and Cellular Biology, SB RAS, Novosibirsk, Russia; 2 Novosibirsk State University, Novosibirsk, Russia; Fralin Life Science Institute, Virginia Tech, UNITED STATES

## Abstract

Morphologically, polytene chromosomes of *Drosophila melanogaster* consist of compact “black” bands alternating with less compact “grey” bands and interbands. We developed a comprehensive approach that combines cytological mapping data of FlyBase-annotated genes and novel tools for predicting cytogenetic features of chromosomes on the basis of their protein composition and determined the genomic coordinates for all black bands of polytene chromosome 2R. By a PCNA immunostaining assay, we obtained the replication timetable for all the bands mapped. The results allowed us to compare replication timing between polytene chromosomes in salivary glands and chromosomes from cultured diploid cell lines and to observe a substantial similarity in the global replication patterns at the band resolution level. In both kinds of chromosomes, the intervals between black bands correspond to early replication initiation zones. Black bands are depleted of replication initiation events and are characterized by a gradient of replication timing; therefore, the time of replication completion correlates with the band length. The bands are characterized by low gene density, contain predominantly tissue-specific genes, and are represented by silent chromatin types in various tissues. The borders of black bands correspond well to the borders of topological domains as well as to the borders of the zones showing H3K27me3, SUUR, and LAMIN enrichment. In conclusion, the characteristic pattern of polytene chromosomes reflects partitioning of the *Drosophila* genome into two global types of domains with contrasting properties. This partitioning is conserved in different tissues and determines replication timing in *Drosophila*.

## Introduction

The polytene chromosomes of dipterans are giant entities showing a distinct banding pattern. Interbands are the most decompacted regions of polytene chromosomes, with a DNA packing ratio of 3–15. According to their morphology, bands can be classified into “grey” and “black.” The former are relatively thin decompacted structures with a DNA packing ratio of 54–63. The latter are normally thicker and more tightly packed, with the DNA packing ratio at times exceeding 200 [1–4]. These bands serve as anchor points on cytogenetic maps of polytene chromosomes and open map sections and subsections [5, 6]. Some of the most prominent black bands are the bands of intercalary heterochromatin (IH). They are the largest among black bands. In salivary gland polytene chromosomes, they fail to complete replication in the S phase of the endocycle and stay under-replicated, leading to special morphological characteristics of these regions, with breaks, constrictions, and ectopic contacts between bands [7]. As demonstrated on a variety of dipteran models, the band/interband pattern is consistent and conserved in polytene chromosomes dissected from diverse tissues [8–12].

Identification of the genomic coordinates of 32 interbands has led to the conclusion that the chromatin organization and protein composition of interbands in polytene chromosomes are conserved across tissues. These interbands are characterized by open chromatin, and many of them correspond to 5' ends of housekeeping genes [4, 13–15]. It has been demonstrated by identification of the genomic positions of 60 IH regions [16, 17] that these bands correspond to extended genomic regions showing low gene density and containing genes with narrow temporal expression patterns. In different cell types, they appear as silent chromatin types, are late replicating, enriched with SUUR and LAMIN Dm0 (LAMIN) proteins, and depleted of proteins and histone modifications that are associated with active transcription [16–18].

The conservation of interband properties in different tissues has enabled the development of an approach for predicting the morphological structures of polytene chromosomes by means of data on the distribution of interband-specific chromatin proteins in cell cultures. On the basis of bioinformatic analysis of this distribution in four cell cultures, Zhimulev and colleagues have divided the *Drosophila* genome into four chromatin types formerly referred to as “cyan,” “blue,” “green,” and “magenta” [4]. These were later renamed to “aquamarine,” “lazurite,” “malachite,” and “ruby,” respectively [19]. In all the four cell lines, aquamarine chromatin is substantially enriched with all the proteins that are typical of polytene chromosome interbands, in particular, interband-specific protein CHRIZ/CHROMATOR [20], “open chromatin” proteins, histone modifications, nucleosome-remodeling complexes, and transcription factors. This chromatin type tends to harbor transcription start sites and 5' untranslated regions of genes. The genes whose promoters are found in the chromatin of this type tend to be expressed in a large number of tissues. Over 90% of ORC2-binding sites are associated with this chromatin type both in cell cultures and in salivary gland polytene chromosomes, indicating the key role of aquamarine chromatin in replication initiation [4]. Lazurite chromatin is not associated with CHRIZ. This chromatin type is rich in proteins involved in transcription elongation and typically encompasses gene bodies [19]. Ruby chromatin is completely devoid of interband-specific proteins, overlaps almost exclusively with repressive chromatin states in different cell lines (according to [21, 22]), and is enriched with LAMIN, SUUR, and proteins associated with Polycomb silencing [4, 18, 19]. Significant enrichment of the insulator proteins CP190, SU(HW), and mod2.2 isoform of mod(mdg4) has been observed in malachite chromatin [18]. Two types of chromatin, aquamarine and ruby, are highly stable across cell lines, that is, in all the four cell lines, they have similar protein composition [19].

Genomic distribution of the four chromatin types is closely associated with polytene chromosome morphology. All the interbands that have been precisely localized to date are associated with the aquamarine chromatin type. At the same time, not all aquamarine segments correspond to interbands [4, 18, 19]. It has been reported that thin grey bands and sometimes small portions of large dense bands can form from lazurite chromatin [4, 23]. As demonstrated in region 10A-B, the zones of alternating lazurite and aquamarine chromatin perfectly follow the pattern of alternating grey bands and interbands on a cytological map [4]. IH bands and large black bands are to a high degree represented by ruby chromatin (80% of the total length) with short malachite insertions (11%) [18]. As revealed in the distal part of chromosome 2R (sections 58 through 60 on the cytological map) as an example, ruby chromatin can serve as a marker of any black band, including quite thin ones [24]. Thus, it has been demonstrated in separate regions that the four-color chromatin model is an effective tool for identification of the genomic coordinates of various morphological structures of polytene chromosomes.

New insights into how polytene chromosome morphology is linked to the organization of the *D*. *melanogaster* genome have recently been gained from analysis of three-dimensional (3D) organization of chromatin [4, 25–28]. For *D*. *melanogaster*, Hi-C data have been published and the boundaries of the regions of intrachromosomal interactions [referred to as topologically associating domains (TADs)] have been defined for cultured cells, whole embryos, and salivary gland cells [26–31]. In accordance with chromatin features within TADs, Sexton and coworkers [30] have split these domains into four classes: active chromatin domains, PcG domains, HP1 domains, and the so-called “null” chromatin domains; the latter are not enriched with any known histone modifications but enriched with histone H1 and LAMIN. The last three domain classes can be regarded as silent. In *D*. *melanogaster*, active TADs are normally short. They are detectable at a mapping resolution of 4–10 kb [29, 30] but not at a Hi-C resolution of 15–20 kb [26, 27]. At resolution 500 bp, active domains are seen as clusters of smaller domains [28]. Chromatin folding differs substantially between active and silent TADs [30, 31]. Evidence exists that partitioning of the genome into these two contrasting TAD types is closely associated with partitioning of the genome into domains highly enriched with housekeeping genes and domains highly enriched with tissue-specific genes [31]. The factors defining TAD boundaries in *D*. *melanogaster* are largely the same as the features typical of interbands [25, 27, 28, 32]. Most TAD boundary sites in *D*. *melanogaster* embryos and cell cultures are located in aquamarine and lazurite but not ruby chromatin. Hence, it has been concluded that TADs may correspond to black bands, whereas TAD boundaries tend not to [4, 27]. A high degree of identity has been observed between IH bands in polytene chromosomes and H3K27me3-containing domains in primary spermatocytes, and these domains in turn have been found to be nearly identical to the prominent TADs revealed in different cell types [31]. Hi-C data on salivary gland polytene chromosomes suggest that at least 95% of IH bands correspond to uninterrupted TADs. Moreover, TAD boundaries in salivary glands have allowed the boundaries of five out of six “black” bands in polytene chromosomes to be predicted [26]. Although Hi-C at 15-kb resolution failed to detect TADs shorter than 75 kb in salivary glands, Eagen and coworkers [26] nevertheless proposed that all the black polytene bands—including the shorter ones, which they estimated to correspond to 70% of DNA in the euchromatic arms—are in fact TADs.

The main marker of IH regions is under-replication in polytene chromosomes. These regions, whether in polytene chromosomes or in cultured cells, are distinguished by the very late completion of replication and the manner in which replication proceeds: from the boundaries toward the center [17, 18, 33]. In cell cultures, the latter property is manifested as a gradient of the replication timing of IH bands from their peripheral to central parts ([18] based on [33]). In polytene chromosomes, these properties are confirmed by direct observation of replication progressing from the periphery of IH bands to their centers in *SuUR*^*ES*^ mutants [17], by the correlation between the under-replication degree and band size [17, 34], and by the correlation between replication completion time and band size [17]. In salivary gland cells, the S phase can be divided into early and late by analysis of the incorporation patterns of labeled DNA precursors, visualized in polytene chromosomes. ^3^H-thymidine labeling patterns specific for different S phase substages in salivary gland polytene chromosomes were first described in the 1950s [12, 35–37]. The first to enter replication are interbands, some light bands, and puffs. These events mark the so-called early discontinuous labeling substage. This substage is followed by continuous labeling, which is when all the chromosomal regions are labeled. Continuous labeling is followed by discontinuous labeling, which is traditionally associated with the late S phase. During continuous labeling, virtually all black bands are labeled and complete replication one by one. In the late S phase, only IH and the chromocenter are labeled [38]. In recent years, the use of fluorescently labeled 5-bromo-2'-deoxyuridine and antibodies to PCNA, a protein marker of replication forks, has increased mapping resolution and revealed that there is no such thing as continuous labeling in salivary gland polytene chromosomes or in polytene chromosomes of pseudonurse cells of *otu*^*11*^ mutants. When replication is seen virtually everywhere along the chromosome arms, the large black bands have not even started, but some regions of lightly packed chromatin have completed replication [39, 40]. Thus, the propensity of thick black bands to be the last to commence replication is conserved in polytene chromosomes across different *D*. *melanogaster* tissues. The order of replication completion by these bands is conserved too, as demonstrated by analysis of replication patterns in polytene chromosomes from different tissues in *Drosophila* [10, 40] and in the mosquito *Anopheles stephensi* [41]. When ^3^H-thymidine is used, analysis of the order of replication completion by bands becomes possible only after ≥40–45 sites on each chromosome arm have been labeled. These loci always contain quite large black bands of polytene chromosomes [7, 38]. What remains unclear is whether the replication delay is a conserved property of all black bands in polytene chromosomes. To answer this question, a thorough analysis of replication patterns in polytene chromosomes using advanced techniques of fluorescent labeling is required.

It has long been thought that IH regions are special genomic regions that are more heterochromatic than the surrounding material of the chromosome arms [42, 18]. Put together, data from the analysis of individual regions of salivary gland polytene chromosomes, the properties of four chromatin types, the organization of chromatin into TADs, and replication timing in polytene chromosomes suggest that all the black bands (that is, the most tightly packed bands) marked by ruby chromatin localize to the genomic regions that tend to possess IH properties. Similar to IH, black bands largely contain tissue-specific genes and chromatin types silent in most tissues; also, they are arranged into TADs and are late replicating in most tissues. The regions between black bands appear as alternating grey bands and interbands. These regions are enriched with housekeeping genes and replication origins; additionally, they are composed of permanently open chromatin and are early replicating. It appears that the patterning of polytene chromosomes into compacted black bands alternating with less compacted grey bands and interbands should reflect partitioning of the *Drosophila* genome into two global types of domains with contrasting properties. For direct testing of this hypothesis, it is necessary to identify the genomic coordinates of all black bands in an extended genomic region.

The aims of this work were to find the genomic coordinates of all black bands on one whole arm of polytene chromosome 2R, to analyze the properties of these bands, to infer their detailed replication schedule, and to compare replication timing profiles between polytene chromosomes and diploid cells.

## Results

### Mapping of black bands on polytene chromosomes, with region 43F-46A of chromosome 2R as an example

We wanted to map all black bands of polytene chromosome 2R, under the following assumptions: 1) all black bands contain ruby chromatin, and 2) any band is flanked by an interband that is located in aquamarine chromatin.

**Fig 1:**
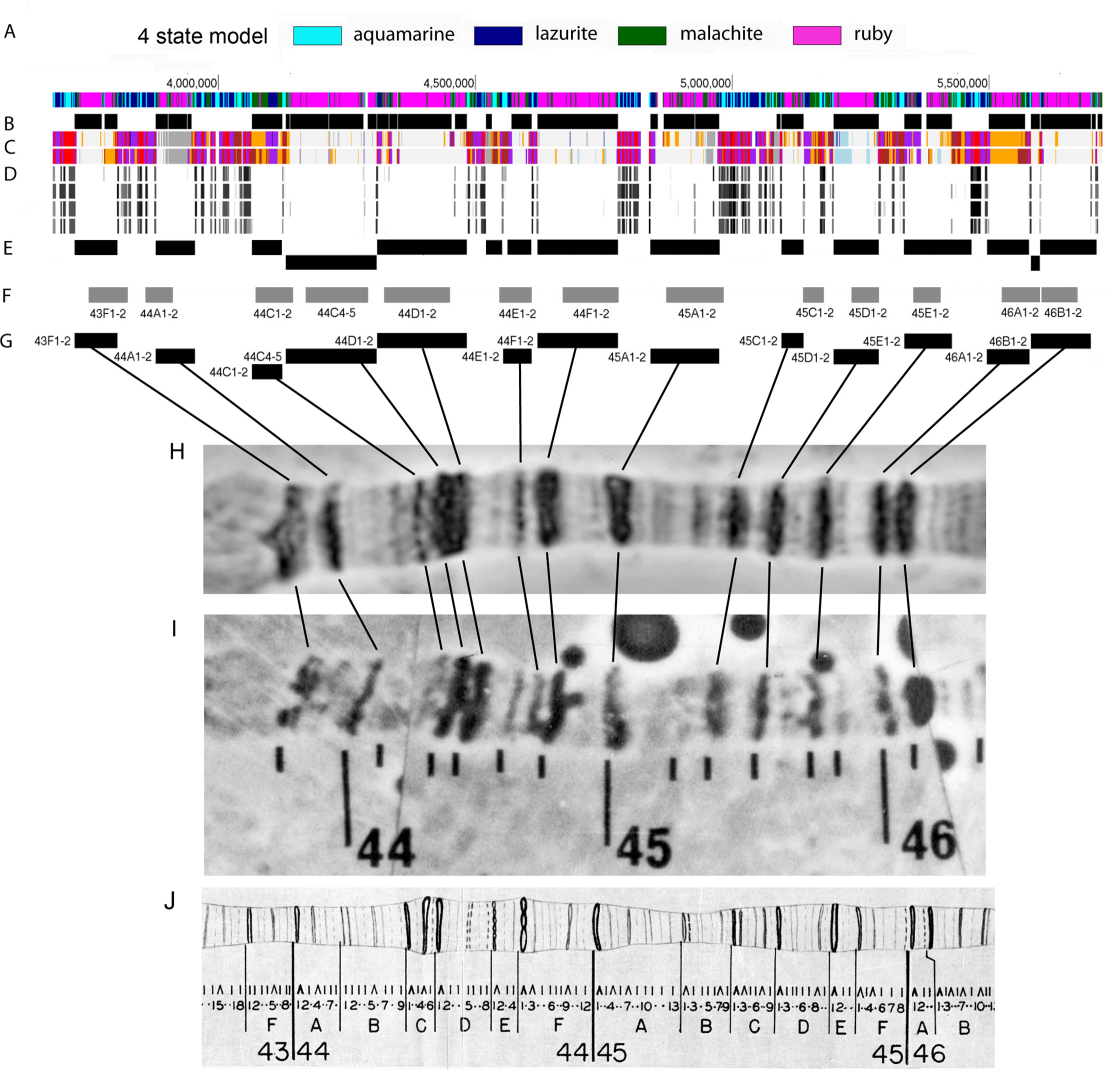
Localization of polytene chromosomes’ black bands on the Drosophila genome map could be predicted by means of data on the distribution of interband-specific chromatin proteins in cell cultures: Region 43F-46B of polytene chromosome 2R as an example. **(A)** Distribution of four chromatin types [4]. Ruby chromatin (depicted in magenta) is devoid of active chromatin markers. Aquamarine chromatin (depicted in cyan) is enriched with all the proteins that are typical of polytene chromosome interbands (see text). **(B)** Locations of condensed bands in polytene chromosomes as predicted from the distribution of ruby chromatin and aquamarine chromatin (each of these bands appears as an interval between aquamarine segments that include ruby chromatin) (see A). **(C)** A distribution of nine chromatin states in S2 cells (top) and Bg3 cells (bottom) [22]. The red color corresponds to chromatin type enriched with active promoters. The presence of this chromatin type in both cell cultures at once is one of the markers of polytene chromosome interbands. **(D)** Enrichment peaks of CHRIZ, the most typical interband protein, in the chromosomes of four cell lines (top to bottom: Bg3, Kc, S2, and Cl8) (modENCODE data). **(E)** Localization of the predicted positions of compacted bands within the framework of the 4-state model combined with the interband criterion filtering (see C, D). **(F)** FlyBase CytoMap locations of compacted bands corresponding to rb-bands (see G) (http://flybase.org). **(G)** Localization of the predicted positions of compacted bands after all correction steps (rb-bands, see text). Bands were assigned names according to mapping data in FlyBase (see text) and Bridges’ detailed map ([47], see J). **(H–J)** Locations of compacted bands in polytene chromosomes from salivary glands (H, J) and pseudonurse cells of *otu*^*11*^ mutants (H, reprinted from [11] under a CC BY license, with permission from Springer Nature: Chromosome Research, original copyright [1995]) in comparison and their correspondence to the bands predicted by the analysis of protein distribution in the cell cultures (G). Compacted black bands as visualized by aceto-orcein staining (H, I) and according to Bridges’ detailed map [47] (J).

To roughly localize ruby-containing polytene chromosome bands (rb-bands) on the genome map, we selected zones that contain ruby chromatin and are flanked by aquamarine chromatin (**Fig 1A and 1B**). Next, we carried out rounds of more detailed localization (**S1 Text**).According to published data, we presumed that all aquamarine segments that contain CHRIZ-binding peaks (according to modENCODE data [43]) in the four cell cultures and overlap with type 1 chromatin (active promoters) within the framework of the 9-state model [22] in both Bg3 and S2 cells always correspond to polytene chromosome interbands. That is why our first step toward better localization was placement of boundaries for the presumed rb-bands only where aquamarine segments had the above-mentioned properties (**Fig 1C–1E**).

To match up the predicted black bands and black bands on Bridges’ map, we proceeded as follows. First, we assigned the predicted genomic fragment to a particular chromosomal region by means of the FlyBase map [44]. Because this map’s resolution allows only the largest bands to be localized (quite roughly) [3], to match up our predictions and particular morphological structures in polytene chromosomes, we used “experimental cytology” data extracted from FlyBase. These data are available for 640 genes of chromosome 2R (**S1 Fig**). To assign names to bands according to the polytene chromosome map, we used Saura’s and Sorsa’s electron microscopic maps [45, 46], on which each visible band is paired with a band on Bridges’ detailed map [47].

When looking for the correspondence between the predicted bands and the bands observed in polytene chromosomes, we introduced the following correction criteria for the locations and boundaries of the predicted rb-bands. When the morphology of the polytene chromosomes was fully consistent with predictions, and the positions of the predicted boundaries did not contradict experimental gene mapping data, the coordinates of the boundary were considered final and an entry was made in **S1 Table**. When the pattern of the predicted bands was not consistent with the morphology or experimental mapping data, we refined the boundaries on the basis of the data about gene expression, TADs [26], distribution of SUUR and H3K27me3 [48] in salivary glands (**S1 Text**). It should be noted that, in each case, the boundary localized to an aquamarine chromatin segment.

Thus, in region 43F-46A of chromosome 2R, we identified 13 genomic intervals (rb-bands) and matched each with one or several bands on Bridges’ map (**Fig 1G**). This region of chromosome 2R stained with aceto-orcein is presented in **Fig 1H**. This staining discriminates between black and grey bands quite well. All the black bands seen in **Fig 1H** correspond to rb-bands that we predicted. Moreover, thicker bands typically map to more extended genomic intervals.

**Fig 1F** shows the genomic locations at which the same bands are found according to the FlyBase map. As readers can see, all the genomic intervals that we mapped as rb-bands in region 43F-46B overlap with the corresponding genomic intervals predicted by FlyBase; however, FlyBase puts some band boundaries rather far from any genomic region that meets the interband criteria (see **Fig 1C and 1D**).

In *D*. *melanogaster*, polytene chromosomes suitable for cytological mapping are present in the pseudonurse cells of *otu*^*11*^ mutants, these cells being germline cells by their origin. **Fig 1I** illustrates the same polytene chromosome region in pseudonurse cells of *otu*^*11*^ mutants (a part of the map from ref. [11]). As in salivary gland polytene chromosomes, black bands in the polytene chromosomes of this type correspond, in terms of size and mutual positioning, very well to the rb-bands that we predicted for the chromosomal region being analyzed.

### Mapping of rb-bands on chromosome 2R and matching them with the FlyBase map

**Fig 2:**
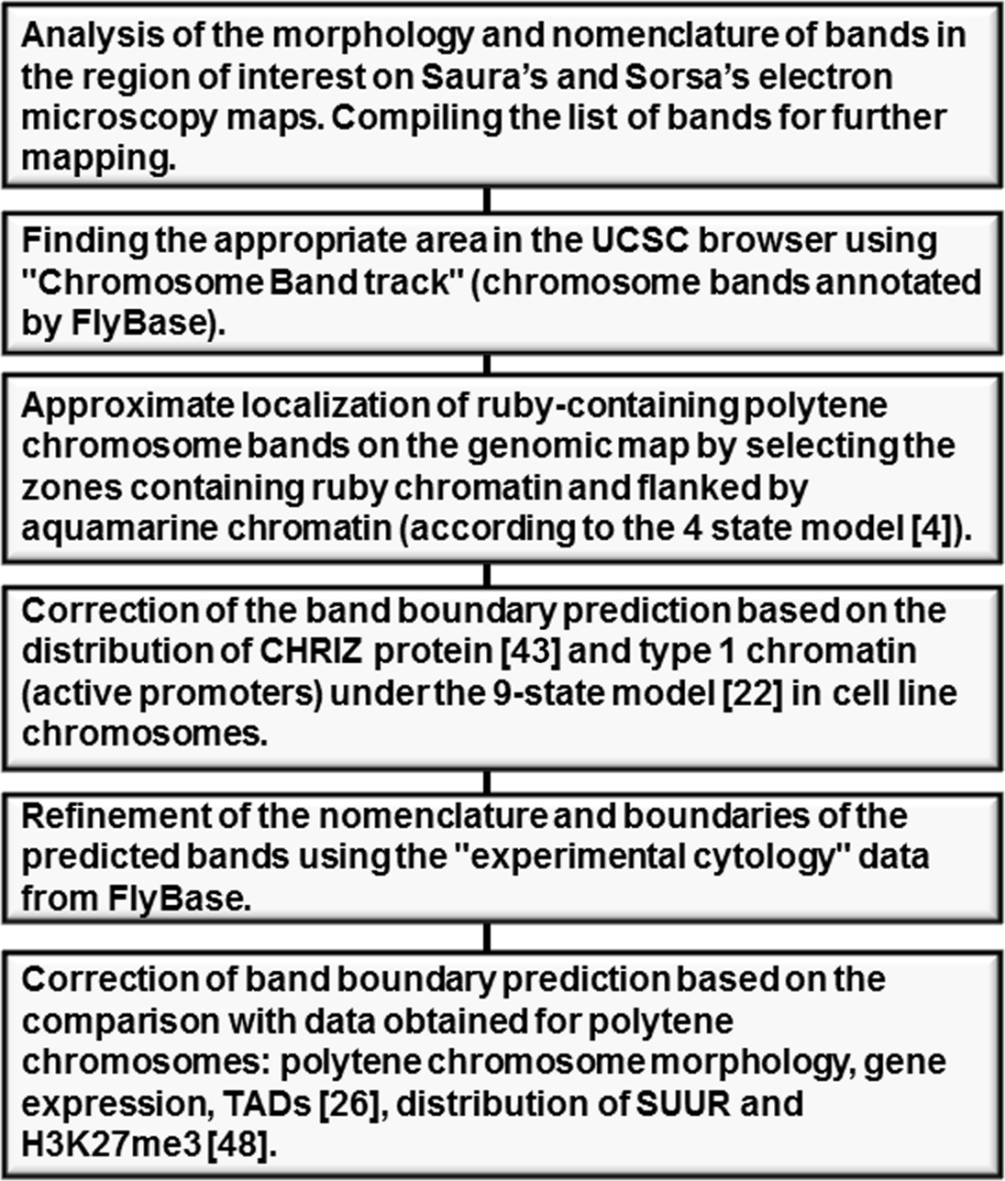
A flow diagram showing the main steps of mapping of black bands.

The genomic coordinates of all rb-bands on chromosome 2R were identified via the approach described above. A flow chart showing the main steps of the approach is given in **Fig 2**. We mapped 159 black bands (ruby bands, rb-bands throughout) (11.6 Mb of DNA in total, or 60% of the genomic region in question) and the intervals between them (INTs throughout) (7.6 Mb of DNA in total, or 40% of the genomic region in question), respectively. The bands varied from 15 to 500 kb in size (**S1 Table**).

To additionally test the correspondence between our predictions and their homonyms on Bridges’ map for accuracy, we used data on *in situ* hybridization of P inserts from the BDGP project [49]. Although the resolution of *in situ* hybridization does not always allow the probe to be localized accurately to a thin band, all the photographs were fully consistent with our mapping (for an example, see **S2 Fig**).

We compared the positions of rb-bands with the FlyBase coordinates of the homonymous bands. The genomic regions occupied by most of our rb-bands (142 out of 159) overlap with FlyBase mapping predictions. Nevertheless, even when bands overlap, FlyBase is seen to grossly misplace band boundaries and to mostly underestimate band sizes. In total, the FlyBase bands contain 30% less DNA than our bands do (9.3 vs. 11.9 Mb). In particular, 80% of the material in the FlyBase bands overlaps with our bands and only 64% of DNA in our bands overlaps with their FlyBase homonyms.

DNA under-replication in polytene chromosomes invariably occurs within bands. This is best seen in electron micrographs of the chromosome breaks in regions of IH [7]. On chromosome 2R, 11 under-replication zones have been identified proximally to 42A [34]. All of them overlap with rb-bands (**S1 Table**). Rb-bands contain 97% of material from the under-replication zones, whereas the FlyBase counterparts contain only 70%. This observation implies that the quality of our mapping is much higher than that of FlyBase.

### Replication timing of 2R rb-bands

**Fig 3:**
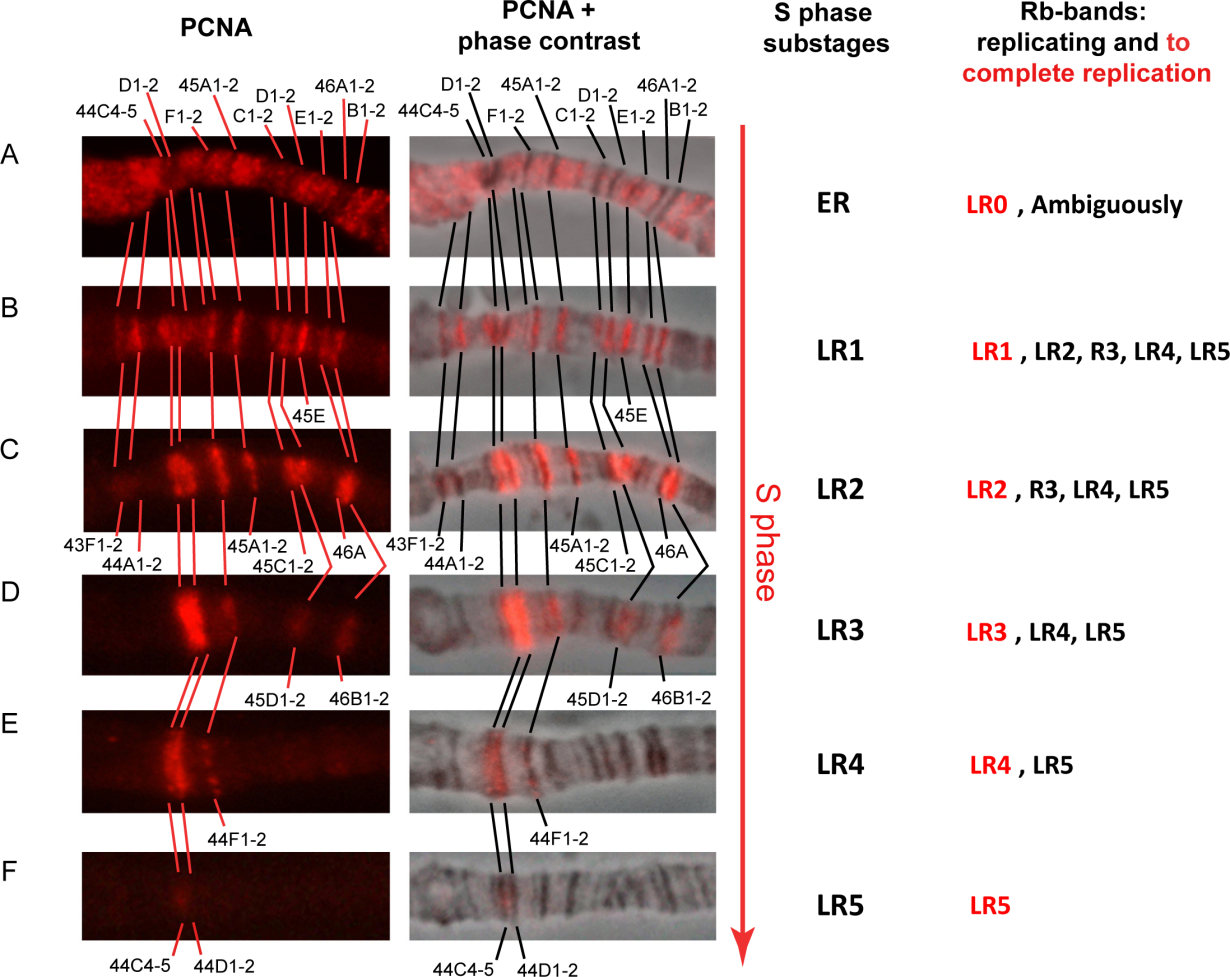
Cytological mapping of the binding sites of anti-PCNA antibodies breaks down the S phase of the endocycle into six substages and assigns rb-bands to replication completion groups. **First column:** The replication pattern of bands in the regions 43F-46B of polytene chromosome 2R as visualized by antibodies against PCNA (red) on *SuUR*^*ES*^ (A) and Oregon R (B–F) polytene chromosomes. Top to bottom: consecutive substages of the S phase, from the earliest (A) to latest (F). (A) “Black” bands not labeled yet. (B–F) Discontinuous labeling. Band designation appears at the substages at which these bands end replication. **Second column:** superimposed images, immunolocalization and phase contrast. **Third column:** Replication pattern-based substages of S phase, where ER is the earliest substage and LR1–LR5 are the subsequent provisionally late substages. **Fourth column:** Each rb-band was assigned to one of five groups, LR1 through LR5, according to the substage at which this rb-band ends replication. A small group of bands with no PCNA at LR1 was classified as LR0. For each substage, all replicating groups and all bands that completed replication are indicated (red text).

**Fig 4:**
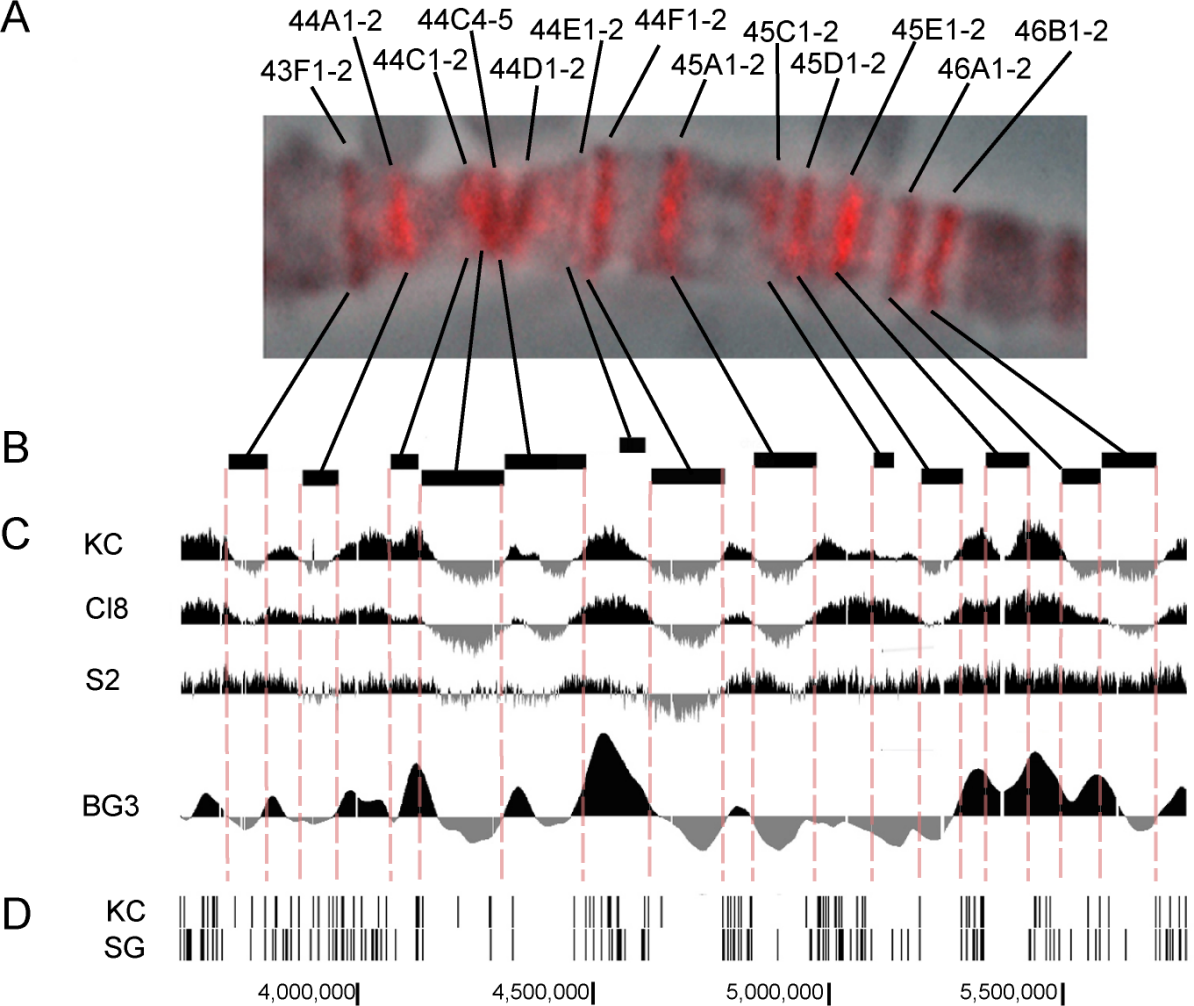
All rb-bands display delayed replication in salivary gland polytene chromosomes and correspond to local minima on replication profiles for cell cultures. **(A)** The replication pattern in salivary gland polytene chromosomes with all rb-bands labeled by PCNA in the region 43F-46B of polytene chromosome 2R as an example. The very existence of a pattern like this suggests that rb-bands are replicated later than the intervals between them. **(B, C)** Locations of rb-bands scaled to the genome map (B) and replication profiles in the cell cultures in comparison: data on KC, Cl8, S2 [33], and BG3 [50] (C) suggest that local minima on replication profiles for the cell cultures fall within rb-bands. This in turn suggests that rb-bands replicate later than their flanking regions. **(D)** Distribution of ORC2 peaks in Kc cells and in salivary glands [51] shows typical zones of low peak density in rb-bands and a prominent ORC2 signal confined to the regions between the rb-bands.

To address replication timing on chromosome 2R, we stained salivary gland polytene chromosomes with antibodies to PCNA. **Fig 3**illustrates some typical staining patterns for region 43F-46B of chromosome 2R in the order of appearance in the S phase. Chromosomes in **Fig 3A** are in the early S phase. Signals are not yet coming from individual bands but are emitted from “zones” composed of groups of grey bands and interbands. Most of the black bands are not yet replicating. The pattern in **Fig 3B** appears to be an inversion of the previous one. All black bands yield bright PCNA signals. The thickest bands, 44C4-5 and 44D1-2, got labeled only peripherally. As the S phase proceeds through its substages, increasingly fewer bands have signals, and at the end of the S phase, only 44C4-5 does (**Fig 3C–3F**). 44C4-5 is devoid of “classic” IH bands, with their under-replication fate, and nevertheless, at the end of the S phase, most nuclei have this weak signal in 44CD. At the substage corresponding to the pattern in **Fig 3B**, virtually perfect correspondence is observed between rb-bands and the bands that get labeled at this substage (**Fig 4A and 4B**). Because the intervals between these bands at this time emit virtually no signals, it can be assumed that all the rb-bands in this particular genomic region complete replication later than the intervals between them.

Next, we analyzed the order in which all the rb-bands of chromosome 2R are replicated (**S3 Fig**). As in 43F-46A, here we identified six distinct PCNA staining patterns, which we designated ER (early replication) and LR1–LR5 (late replication 1–5; **Fig 3 and S3 Fig**). Each band listed in **S1 Table** was assigned to one of the substages (LR1–5), meaning that this is the substage at which the band completes replication. The bands that had completed replication before LR1 were assigned to LR0.

The main difference of our approach from the approach described in the group of papers where replication was mapped [12, 35–38] is that we did not attempt to assign the coordinates to a single replication signal that may capture several adjacent bands simultaneously. On the contrary, we first identified a band, named it and assigned coordinates, and then tried to match it with the stage at which it finishes replication, that is, the stage at which it does not yield any PCNA signals. Replication substage–specific lists of rb-bands and lists of bands qualifying as late replicating by different criteria in the literature are given in **S2 Table** for comparison. The table shows that seven of 18 LR5 bands are found among the IH regions mapped genome-wide by Belyaeva and coworkers [17], 11 occur in the under-replication zones identified by Yarosh & Spradling [34], three have breaks in the polytene chromosomes of wild-type larvae [7], and all have breaks in the lines with additional doses of the *SuUR* gene [38].

Additionally, the LR5 bands correspond nearly perfectly to SUUR-binding sites in wild-type lines [38, 52]. At substage LR4, 12 more bands are added to the list. Under-replication in wild-type lines has been described for only two of these 12 [17, 34]. Most of these bands have breaks in the lines with additional doses of the *SuUR* gene. All these bands have been described as SUUR-binding sites [38]. The bands that complete replication at LR3 (17 in total) are never under-replicated: not even in the lines that carry additional doses of *SuUR*. Zhimulev and coworkers [38] recognized six of them as being late replicated and most of them, 14, as binding SUUR in the lines with additional doses of *SuUR*. The late-replication lists include only a few LR2 bands and none of the LR1 or LR0 bands. Thus, most of the bands that are recognized as late replicating by various researchers were found to be LR3–LR5.

It can be concluded that our replication timing is in good agreement with the data published previously and generalizes them. The later our rb-bands complete replication, the more criteria for late replication they meet. The latest-replicating bands coincide well with IH regions, that is, with under-replication regions. Nevertheless, any segregation of regions into early and late replicating is rather provisional because it always depends on which criteria were chosen.

### Replication timing of rb-bands is similar among different cell types

**Fig 5:**
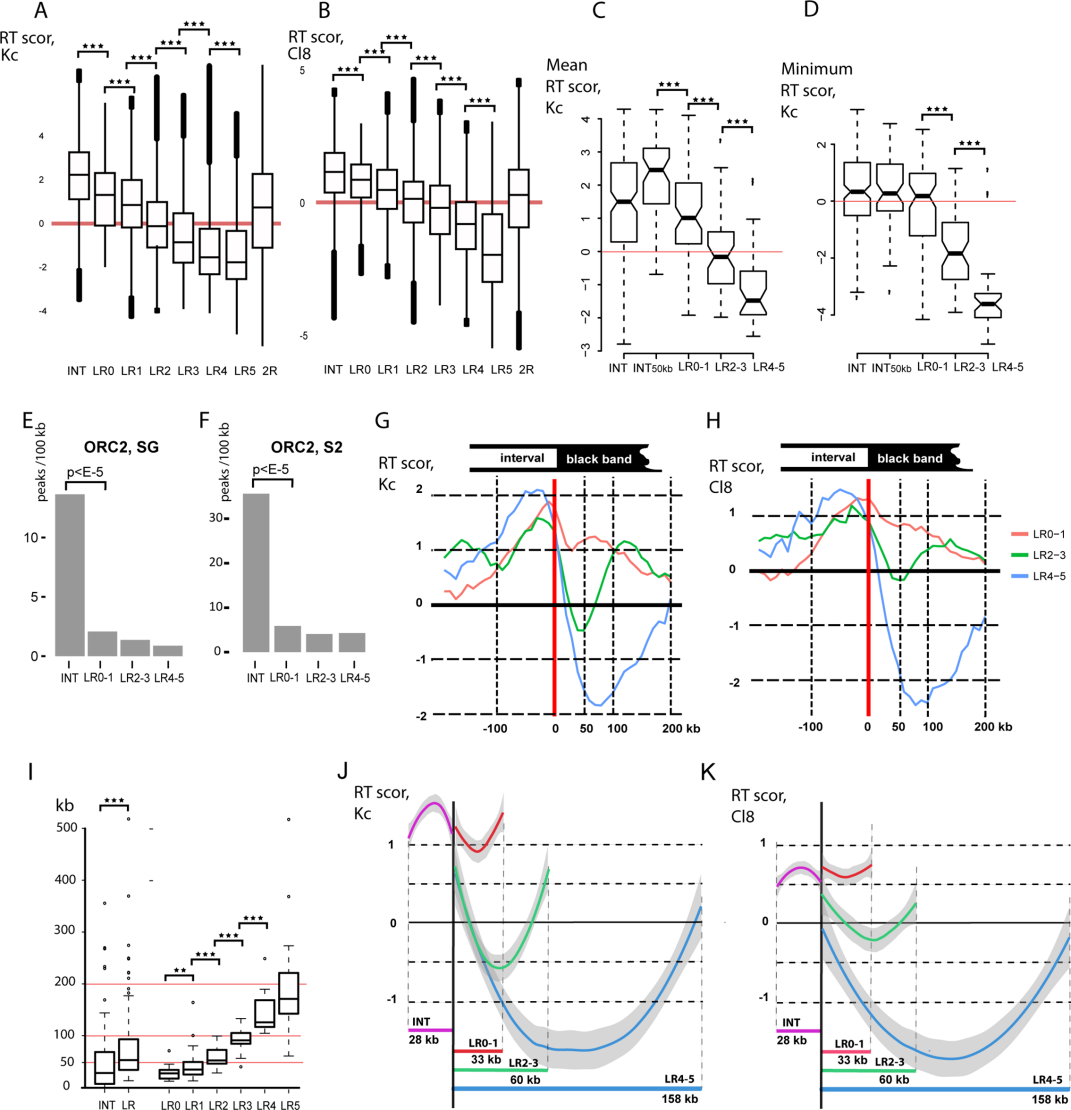
Similarity in replication timing between polytene and diploid cells. To infer replication timing in polytene chromosome 2R, all mapped rb-bands were rated based on their replication completion time in salivary gland polytene chromosomes (LR0–LR5, where LR5 are the last to complete replication, see text and Fig 2). Intervals between rb-bands (INTs) in salivary gland polytene chromosomes complete replication before any rb-band does. Data on replication timing for chromosome 2R DNA sequences in cell cultures according to [33]. **(A,B)** Boxplots of replication time scores in Kc (A) and Cl8 (B) cells for all DNA sequences corresponding to INTs, LR0–LR5 bands, and the entire fragment being studied on chromosome 2R. All groups of rb-bands are significantly different from INTs and from one another (the Mann–Whitney *U* test, p < E-100). A tendency is clear: the later bands complete replication in salivary gland polytene chromosomes, the later are the corresponding DNA sequences replicated in cell cultures. On the *y*-axis: replication time scores according to [33], with +6 corresponding to early replication, and −5 to late replication. (**C,D)** For each rb-band and for each INT, the mean (C) and minimum (D) replication time scores in Kc cells were calculated according to [33]. In the boxplots: the distribution of the mean (C) and minimal (D) scores for all INTs and the rb-bands assigned to three groups (LR0–1, LR2–3, and LR4–5) in accordance with replication completion time in salivary gland polytene chromosomes. All intervals (INTs) and intervals longer than 50 kb (INT50kb) were analyzed separately. Mean replication time in Kc cells is significantly different between band groups rated based on replication completion time in salivary gland polytene chromosomes (C). Replication completion time in Kc cells is significantly different among LR0–1, LR2–3, and LR4–5 bands (C). ***p < E-5 (Mann–Whitney *U* test). On the *y*-axis: replication time scores according to [33] as in C. **(E, F)** Density of ORC2 peaks (peaks/100 kb) (according to [51]) in salivary glands (E) and S2 (F) cells. **(G,H)** Mean replication time scores in Kc (G) and Cl8 (H) cells for all sequences within 1-kb windows at the same position relative to the band boundary (red vertical line) in all LR0–1 (red curve), LR2–3 (green curve), and LR4–5 (blue curve) bands. Right-hand boundary data were mirrored and combined with left-hand boundary data. Within-band regions are to the right of the border; outside regions are to the left. On the *x*-axis: distances from the border. On the *y*-axis: replication time scores according to [33]. **(I)** Delays in replication completion in salivary gland polytene chromosomes correlate with band lengths. **p < E-2, ***p < E-5 (Mann–Whitney *U* test). On the *y*-axis: sizes of rb-bands (LR, LR0–LR5) and intervals between them (INTs) in kb. **(J,K)** Mean replication time scores in Kc (G) and Cl8 (H) cells for rb-bands and INTs normalized to the median band size in each group (28 kb for INTs, 33 kb for LR0–1, 60 kb for LR2–3, and 158 kb for LR4–5). On the *y*-axis: replication time scores according to [33]. Grey lines denote 95% confidence intervals. (G, H, J, K) The U-shape of averaged replication profiles within bands suggests that replication proceeds from the boundaries to the center. Deeper profiles correspond to later-replicating bands in polytene chromosomes (LR0–1 < LR2–3 < LR4–5), suggesting that these bands will complete replication in Kc cells later.

Once we profiled the replication timing of rb-bands, we could compare replication timing between polytene chromosomes and diploid cells. **Fig 5A and 5B** shows boxplots of replication timing in Kc and Cl8 cells according to Schwaiger and coworkers [33] for all DNA sequences within genomic intervals corresponding to LR0–LR5 bands and INTs. As presented in the figure, the rb-bands in polytene chromosomes follow the same order of replication as the corresponding DNA sequences in the cell cultures. In the cell cultures, INTs complete replication on average earlier than rb-bands. Furthermore, in Kc cells, we determined the mean replication timing of all DNA sequences in individual bands and INTs. INTs longer than 50 kb were given special consideration (**Fig 5C**). On average, these INTs were replicated significantly earlier than the rb-bands. The time of replication completion was also significantly different among LR0–1, LR2–3, and LR4–5 bands. The rb-bands that are the last to complete replication in polytene chromosomes are the last to complete replication in Kc cells (**Fig 5D**). It can be concluded that, at the level of rb-bands and intervals between them, chromosomal regions are replicated in the same order in polytene chromosomes as in cell cultures.

### Local minima in replication profiles correspond to rb-bands

A visual comparison of the rb-bands and the replication profiles published previously for the four cell cultures (**Fig 4B and 4C**) indicates that in Kc and BG3 cells, the portions of the replication curves corresponding to rb-bands assume a U-shape, suggesting that the central part of each band is replicated considerably later than its peripheries. Moreover, most rb-bands were found to have positive replication scores at their peripheries, while the bulk of each band has a negative score. A similar tendency, albeit less pronounced, is seen in the Cl8 and S2 profiles.

These data are consistent with the fact that rb-bands coincide well with the zones that are virtually devoid of ORC2-binding peaks in Kc cells and in salivary glands (data were taken from [51], **Figs 4D, 5E and 5F**). Comoglio and Paro [53] mapped replication initiation sites in S2 and Bg3 cells by sequencing newly synthesized DNA. According to that work, INTs too contain more replication initiation sites than rb-bands do, although the difference between INTs and rb-bands is not so great as that for ORC2 (**S4 Fig**).

We generated an averaged replication profile for the regions surrounding bands from each of three groups (LR0–1, LR2–3, and LR4–5) in Kc and C18 cells (**Fig 5G and 5H and S5 Fig**). In all three cases, a gradient of the replication score from the periphery to the center is seen, with the minimum at ~75 kb for LR4–5, at ~50 kb for LR2–3, and at less than 50 kb for LR0–1. This behavior of the averaged replication profile is consistent with size differences between bands in different groups (**Fig 5I**). The coefficients of correlation between band size and the mean replication score corresponding to this band were found to be -0.47 (p < E-10) for Kc cells and -0.51 (p < E-10) for C18 cells.

It can be concluded that the local minima in the replication profiles for diploid cells fall within rb-bands, while the local maxima fall outside them, although quite close to their boundaries. This replication profile is consistent with the theoretically predicted U-shaped profile, where the region deprived of early origins is flanked by extended replication initiation zones [54]. This conclusion is well supported by plots where the replication score data are normalized to the median length of rb-bands in groups LR1–0 (red profile), LR2–3 (green profile), LR4–5 (blue profile), and INTs (purple profile; **Fig 5J and 5K**).

### Rb-bands and INTs differ in gene density, expression, and tissue specificity

**Fig 6:**
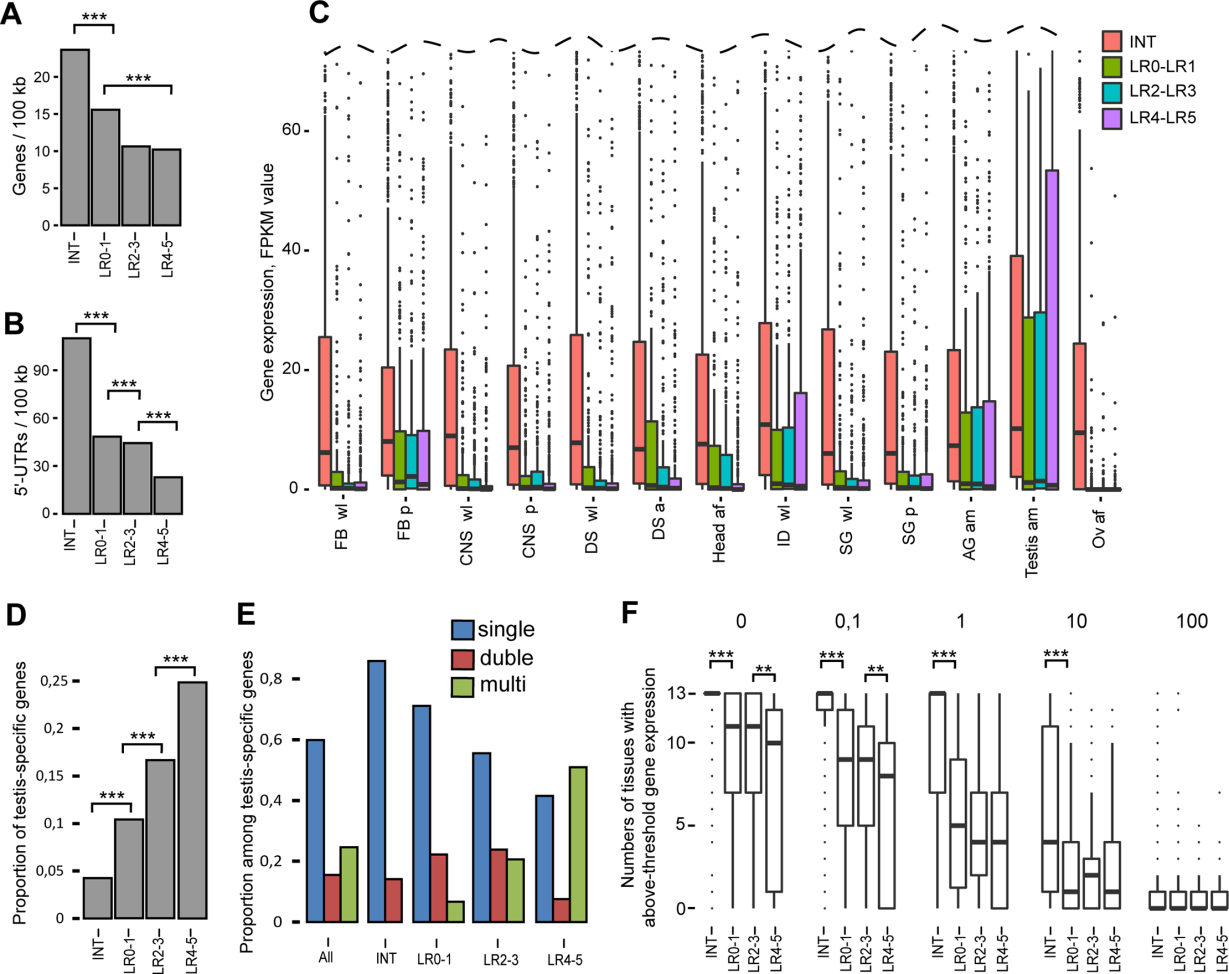
The rb-bands and INTs differ from each other in gene density, expression and tissue specificity. **(A, B)** Rb-bands in the three replication completion groups (LR0–1, LR2–3, and LR4–5) are significantly depleted in genes as compared with intervals between these bands (INTs). (A) Gene density (genes per 100 kb) according to FlyBase annotations and (B) 5'-UTR density (5'-UTRs per 100 kb). Two-sample permutation tests, 10,000 iterations, ***p < E-5. **(C)** The expression of genes lying entirely in rb-bands in various tissues is significantly different from that of genes lying entirely in the intervals between rb-bands. Boxplots of gene expression data for LR0–LR1 (green bars), LR2–LR3 (blue bars), and LR4–LR5 (purple bars) bands and intervening sequences (red bars) in different larval or adult tissues. Gene expression data in FPKM (Fragments Per Kilobase Million) were generated by modENCODE ([43], March 2016 data freeze). All groups of rb-bands are significantly different from INTs and from one another (the Mann–Whitney *U* test, p < E-26). **FB wl**: fat bodies, wandering larvae; **FB p**: fat bodies, 2-day prepupae; **CNS wl**: central nervous system, wandering larvae; **CNS p**: central nervous system, two-day old prepupae; **DS wl**: digestive system, wandering larvae; **DS a**: digestive system, one-day old mixed adults; **Head af**: heads, one-day old female adults; **ID wl**: imaginal discs, wandering larvae; **SG wl**: salivary glands, wandering larvae; **SG p**: salivary glands, prepupae; **AG am**: male accessory glands, male adults; **Testis am**: testes, male adults; **Ov af**: female gonads, female adults. **(D)** The later the bands are replicated, the higher the proportion of testis-specific genes they tend to have. The proportion of testis-specific genes (according to [55]) in INTs and rb-bands grouped based on their replication completion time (LR0–1, LR2–3, and LR4–5). Two-sample permutation tests, 10,000 iterations, ***p < E-3. **(E)** The latest-replicating rb-bands are typically enriched with multigene clusters of testis-specific genes. The proportion of stand-alone testis-specific genes, two-item, and multiple-item clusters among all testis-specific genes (according to [55]) on chromosome 2R (All), in intervals between rb-bands (INTs) and in rb-bands classified based on replication completion time in salivary glands (LR0–1, LR2–3, and LR4–5). Blue bars correspond to the proportion of stand-alone testis-specific genes among all testis-specific genes; red bars, to the proportion of two-item clusters; green bars, to the proportion of multiple-item clusters. The INTs are devoid of multigene clusters of testis-specific genes. This distribution is significantly different from random (resamples n = 10,000, goodness-of-fit test, p < E-5.). LR4–5 rb-bands are enriched with multigene clusters and depleted of stand-alone testis-specific genes. This distribution is significantly different from a random one (resamples n = 10,000, goodness-of-fit test, with provision for cluster size, p < E-5). **(F)** Genes in the rb-bands tend to be expressed in a limited number of tissues. By contrast, the intervals between rb-bands are enriched with genes that are highly expressed in most of the tissues under consideration. Boxplots of the numbers of tissues with above-threshold gene expression. Threshold values: 0; 0.1; 1; 10; 100. At 0–10, INTs significantly differ from any rb-band (***p < E-30). At 0 and 0.1, LR2-3 bands significantly differ from LR4–5 bands (**p < E-5; Mann–Whitney *U* test). Gene expression data were generated by modENCODE [43].

We analyzed gene density in the LR0–1, LR2–3, and LR4–5 rb-bands and INTs. As **Fig 6A and 6B** indicates, all rb-bands show significantly lower densities of 5' untranslated regions (UTRs) and entire gene loci than INTs do.

We analyzed gene expression in LR0–LR5 bands and INTs from 13 larval and adult tissues. As illustrated in **Fig 6C**, all the LR0–LR5 rb-bands show lower median expression levels in all tissues in comparison with INTs. The gene expression profiles look similar in all tissues, except for the testes, in which a relatively large group of genes manifested very high expression. This finding is consistent with the fact that male-specific genes represent a considerable proportion of genes in under-replication regions [16] and black bands (**Fig 6D**). Incidentally, the genes in the bands are virtually not expressed in the ovaries.

A work by Shevelev and coworkers provides a list of testis-specific genes in *D*. *melanogaster*, some of them grouped into multigene clusters [55]. The region being analyzed on chromosome 2R contains 284 of the listed genes, of which 70 are in 16 clusters with more than two testis-specific genes in each and 44 are in two strong clusters. Analysis of the distribution of testis-specific genes and their multigene clusters in INTs and rb-bands shows that all the genes in these 16 clusters lie entirely within rb-bands: 12 in LR4–5, three in LR2–3, and one in LR0–1. That is, all the clustered genes are in rb-bands, whereas stand-alone testis-specific genes are enriched in INTs (**Fig 6E**). Notably, it is the most late-replicating LR4–5 rb-bands that are enriched with multigene clusters among the others. It is noteworthy that all the genes in each cluster lie entirely within one (and only one) rb-band. This distribution is significantly different from random (resamples n = 10,000, goodness-of-fit test, with provision for cluster size, p < E-5). According to the cytological map in FlyBase, four of these 16 clusters fall within intervals between late-replicating bands. There are eight clusters and each corresponds to more than one band, with intervals between them. This is another occasion to compare our mapping with that in FlyBase and to stress that ours much more precisely reflects the functional organization of the *Drosophila* genome.

For each gene, we then identified how many tissues out of the 13 express this gene beyond each preset threshold level (0, 0.1, 1, 10, and 100). As **Fig 6F** shows, at 0, most INT genes are expressed in all 13 tissues. At 0.1 and 1, the median value is equal to 13 too. In rb-bands, however, the median values are substantially lower. It can be concluded that the INTs are enriched with the genes that are expressed ubiquitously at relatively high levels, whereas the rb-bands are enriched with genes that are expressed at lower levels and in fewer tissues.

Next, we checked whether there were differences between the genes in rb-bands and INTs by means of Gene Ontology (GO; **S3 Table**). According to FlyBase, the fragment being analyzed on chromosome 2R contains 2753 protein-coding genes. GO annotations [56] were available for 2408 genes. A total of 982 genes are in LR0-5 bands entirely. GO annotations were available for 817 of them. These genes are enriched with tissue-specific GO terms [57]: sensory perception of chemical stimulus, detection of chemical stimulus, defense response to gram-positive bacterium, and reproduction. The products of the genes in rb-bands perform specific molecular functions: odorant binding, endopeptidase activity, peptidase activity, structural constituent of cuticle, and some others. The bands are enriched with the genes whose products are found on the cell surface or outside cells; this finding additionally characterizes these genes as being tissue-specific. A total of 1470 genes lie within INTs entirely. Among them, 1286 are annotated in GO. These genes were found to be enriched with ~100 GO terms, many of which are related to “regulation of biological process,” “cellular response to stimulus,” and “cellular metabolic process.” The tendency of genes associated with different GO terms to be specifically distributed between bands and intervals lends support to the view that INTs are likely to contain the bulk of housekeeping genes, while tissue-specific genes preferentially localize to bands.

### Rb-bands are significantly enriched with repressive chromatin types and show a sharp change in chromatin protein density at their peripheries

**Fig 7:**
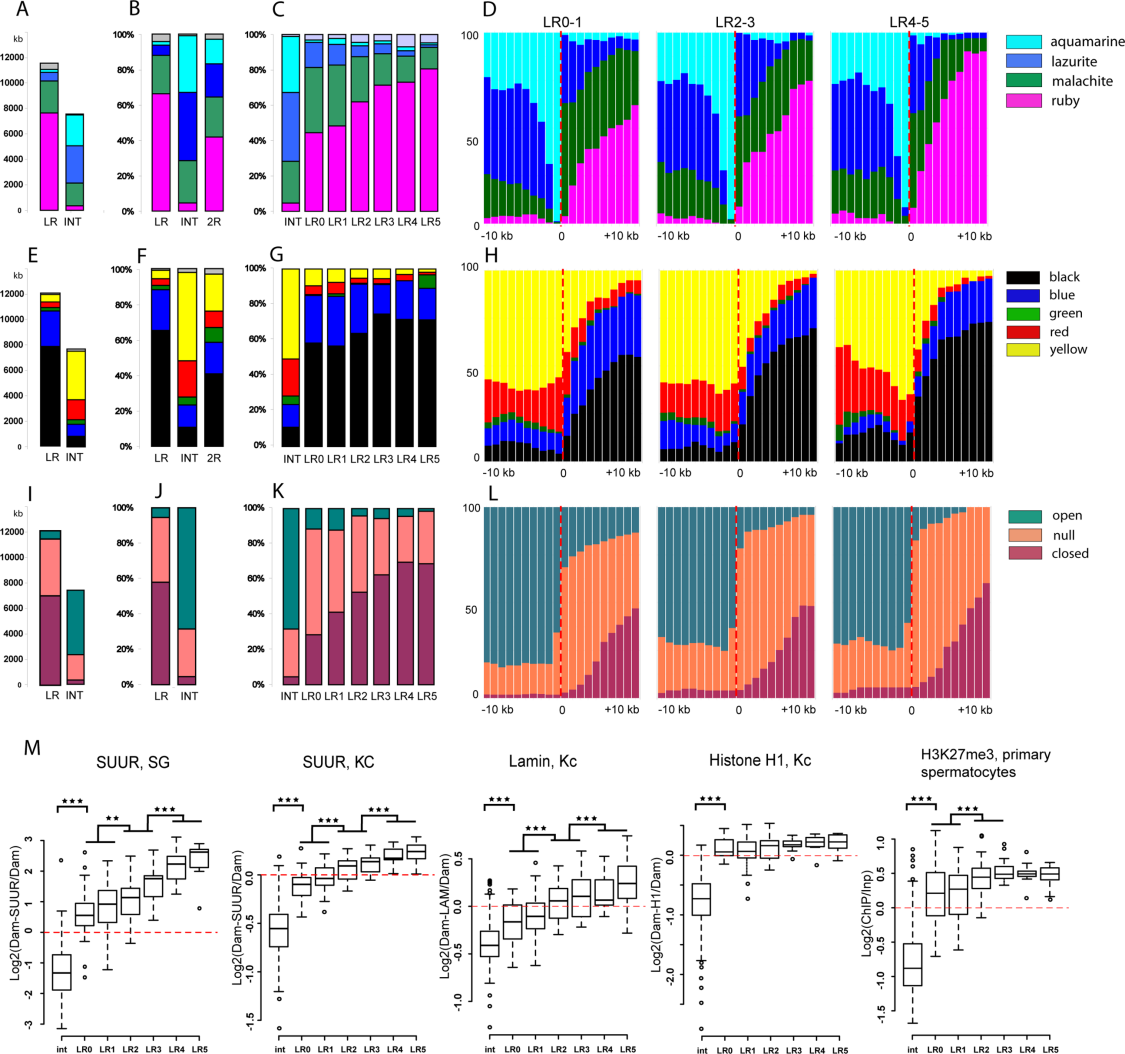
The rb-bands are significantly enriched with repressive chromatin types. **(A)** Distribution of four chromatin types [4] in all 2R rb-bands and INTs. The data are expressed in kb. **(B)** Distribution of four chromatin types [4] in all 2R rb-bands and INTs. The data are expressed in percentage points. **(C)** Distribution of four chromatin types [4] in rb-bands classified into replication timing groups LR0–LR5 and into INTs. **(D)** An averaged distribution of four chromatin types [4] at locations close to the boundaries of LR0–1, LR2–3, and LR4–5 rb-bands. **(E–G)** A distribution of five chromatin types in Kc cells [21] in rb-bands and INTs. The data are expressed in kb (E) and in percentage points (F, G). An averaged distribution of five chromatin types in Kc cells [21] at locations close to the boundaries of LR0–1, LR2–3, and LR4–5 rb-bands. **(I–K)** Distribution of differently compacted chromatin types in S2 cells [58] in rb-bands and INTs. The data are expressed in kb (I) and in percentage points (J, K). **(L)** An averaged distribution of differently compacted chromatin types in S2 cells [58] at locations close to the boundaries of LR0–1, LR2–3, and LR4–5 rb-bands. **(D, H, L)** The band boundary is at position zero. To the right: within-band regions; to the left: outside regions. The increment is 1 kb. **(M)** Boxplots of the distribution of mean scores for repressed-chromatin markers in LR0–LR5 rb-bands and INTs. Markers: SUUR in salivary glands (according to [59]), SUUR in Kc cells [21], LAMIN in Kc cells [60], histone H1 in Kc cells [21], and H3K27Me3 in primary spermatocytes [31]. All rb-bands are significantly different from INTs (***p < E-14, **p < E-3; Mann–Whitney *U* test).

We named our bands rb-bands because all of them contain ruby chromatin. Ruby makes up 42% of DNA in the genomic region in question (2R without the proximal region). Our band prediction technique initially indicated that ruby chromatin is always confined to rb-bands. Nonetheless, only those predictions were included in the final table that could be assigned to a particular cytological structure (a band or a group of bands). Thus, ~4% of ruby chromatin was outside our bands (**Fig 7A and 7B**). An average rb-band is 66% ruby, 22% malachite, 6% lazurite, and 2% aquamarine. The rb-bands contain most of unannotated fragments that offer no predictions according to the 4-state model. The prevalent chromatin types in INTs are aquamarine (32%), lazurite (39%), and malachite (23%; **Fig 7A and 7B**). If we look at the proportions of these four chromatin types in the LR5–LR0 bands, then we will see the following tendency: the later the bands are replicated, the higher is the percentage of ruby chromatin in them (**Fig 7C**). Examination of the distribution of chromatin types in all rb-bands (**S6A Fig**) and the averaged distribution of chromatin types around the boundaries of the LR0–1, LR2–3, and LR4–5 bands (**Fig 7D and S7A Fig**) suggests that lazurite largely localizes to the edges of the rb-bands and does not occur in them deeper than 10 kb from the provisional boundary. In all the three late-replication groups, the most prevalent chromatin type deeper than 5 kb from the provisional boundary is ruby. At 1–2 kb outside each band, the most prevalent chromatin type is aquamarine, in line with our boundary criterion.

Next, we analyzed the distribution of five chromatin types in the rb-bands of Kc cells [21]. The rb-bands are largely composed of black and blue chromatin (**Fig 7E–7G**). Most bands consist of alternating blue and black chromatin domains. Among the 159 bands, only 14 do not include black chromatin (**S6B Fig**). A large number of rb-bands have, in their boundary regions, some open-type yellow chromatin, with its average distribution there being identical to that of open-type lazurite chromatin (compare **Fig 7H** and **S7B Fig** with **Fig 7D**). Intervals in turn appear as alternations of different chromatin types, with open-type yellow and red as the commonest varieties (**Fig 7E and 7F, S6B and S7B Figs)**.

A comparison of rb-bands with the distribution of three chromatin types notable for compactness in S2 cells [58] showed that the bands are substantially covered by tightly packed and intermediate chromatin types. The later the bands are replicated, the higher is the fraction of tightly packed chromatin that they tend to have (**Fig 7I–7L**). A glance at **S6C and S7C Figs** reveals that the most tightly packed chromatin tends to be located near the central parts of the bands, whereas the intermediate types in the boundary regions and shorter bands.

We analyzed the mean SUUR score in salivary glands (DamID data, [59]) for LR0–LR5 bands and INTs. As readers can see in **Fig 7M**, 1) rb-bands are enriched with SUUR in all groups; 2) the later the bands are replicated, the higher is their SUUR score; 3) INTs are virtually devoid of SUUR. The averaged SUUR distribution profile for salivary glands and Kc cells (**S8 Fig**) shows a sharp increase from a minimum at the boundary of the band to a maximum deeper into the band.

The mean LAMIN score (DamID data, [60]) too is higher in all band groups than in INTs (**Fig 7M)**. The following tendency was observed: the later the band is replicated, the higher the LAMIN score it has. The averaged LAMIN distribution profile in the boundary regions of the LR4–5, LR2–3, and LR0–1 bands (**S8 Fig**) shows a sharp increase, and the latest-replicating bands have the highest LAMIN scores. The genomic region being studied is reported to include 67 lamina-associated domains (LADs) [60]. Only two of them do not overlap with any of the rb-bands. There are 50 LADs that overlap with one rb-band each; ten with two; and five with three. As many as 64% of LADs are within bands entirely. All late-replicating bands (LR4–5), except for 50A1-4, overlap with LADs (see **S4 Table**). Seventy percent of LR3, 60% of LR2, and 30% of LR0–1 bands overlap with LADs. Thus, LADs lie in the longer bands, although not all of the shorter, earlier-replicating bands correspond to LADs. It can be concluded that LAMIN is a component of chromatin in all rb-bands, but its overall low binding values and small band sizes make the corresponding regions to meet the statistical criteria used to define LADs.

An averaged distribution profile for yet another component of silent chromatin types, histone H1, in Kc cells (DamID data. [21]) revealed its higher occurrence in rb-bands as compared to INTs (**Fig 7M)** and displays a sharp increase on the band/interband boundary (**S8 Fig**).

Recently, El-Sharnouby and coworkers [31] applied a Hidden Markov Model to the primary spermatocyte H3K27me3 profile and partitioned the genome into two states: (1) depleted (D) domains characterized by very low H3K27me3 content and containing housekeeping genes and their regulators; and (2) enriched (E) domains containing moderate-to-high levels of H3K27me3 and associated with regulated genes, no matter whether active or inactive. We can see that over 90% of rb-band material overlaps with E domains. As **S8 Fig** shows, the behavior of protein distributions and the profiles of replication on D/E domain boundaries match up perfectly with those on the rb-band/INT boundaries. On the other hand, the averaged profile of the H3K27me3 distribution in primary spermatocytes shows a typical sharp increase on the rb-band/INT boundary (**S8 Fig)**. These results point to a considerable match between rb-bands and E domains, in agreement with the prediction made from comparison of E domains and IH bands [31].

### Rb-bands correspond to TADs

We compared rb-bands and salivary-gland TADs [26]. TADs cover 50.5% of the total length of the region being studied on salivary gland chromosome 2R, whereas bands cover 61.1%. As much as 85% of the total TAD length coincides with rb-bands. A total of 61 TADs were identified in the study region of salivary gland chromosome 2R, among which only two did not overlap with rb-bands (**S4 Table**). Many of the TADs correspond to groups of closely spaced bands (band coverage >90%): only as few as 32 TADs overlapped with one rb-band each, and 27 with 2–4 bands (regardless of how many of these rb-bands are in fact groups of closely spaced bands). Examination of band pairs (for example, 44C4-5/44BD1-2 and 46A1-2/46B1-2 in **S9A Fig** and 56A1-2/56B1-2 and 57B2-3/57B4-6 in **S10A Fig**) indicated that the bands lie within a single TAD; however, the Hi-C interaction map (heatmap) shows a distinct “barrier” that partitions TADs into smaller TADs. Electron microscopic section of polytene chromosomes reveals multiple fibers of tightly packed material in the paired bands (**S10A Fig**). This result is consistent with a relatively high probability of contacts within a single TAD according to the heatmap.

Any rb-band longer than 85 kb had more than a 70% overlap with TADs (**S4 Table**). At the same time, most of the shorter bands did not overlap with TADs. The only explanation we can think of is that the mapping of TADs in salivary glands was performed at too low resolution to resolve TADs shorter than 75 kb.

Visual comparison of rb-bands and the salivary gland Hi-C interaction map revealed that not only the longest but also some shorter rb-bands corresponded to the triangles on the heatmap, which indicate the regions with a high probability of internal interactions in them (**S9A Fig** for the region 43F-46B, **S1 Fig** for all 2R). Between them, there are zones where short-range interactions predominate. The predominance of interactions at distances shorter than 1 bin (1 bin amounts to 15 kb) makes all the points along the heatmap diagonal in the intervals between ruby bands look very dark. Their darkness makes them easily distinguishable from the bins that fall into ruby bands. The boxplot in **S9B Fig** supports the view that the distribution itself of contact probabilities along the diagonal (that is, probabilities of short-range interactions) is quite a marker of the 3D chromatin organization and allows the genomic coordinates of large black bands in polytene chromosomes to be predicted. As readers can see in the boxplot, this distribution in rb-bands is similar to that of TADs, whereas INTs resemble interTADs (**S9B Fig**). This is an impressive piece of indirect evidence that all rb-bands correspond to TADs.

In region 46F, the morphology of polytene chromosomes varies: it sometimes features a group of prominent black bands and sometimes a series of decompacted bands on the left of the large prominent band 47A1-2 (**S10B Fig**). This variability can probably be due to the *Hr3* gene, with its high expression shortly before the prepupal stage (according to modENCODE). The heatmap interval corresponding to this variable region revealed alternations of bins with different properties and looked contrasting against extended regions with relatively uniform patterns of interactions. This observation provides further support to the notion that salivary-gland Hi-C allows various morphological features of polytene chromosomes—black bands, intervals between them, and morphologically complex regions—to be predicted.

It has previously been demonstrated that the 3D chromatin organization corresponding to TADs is well conserved across cell types. A visual comparison of rb-bands and the heatmaps generated for interactions along chromosomes 2R in different cell cultures suggested that all our bands correspond to the triangles on the heatmaps that indicate regions with a high probability of internal interactions in them (**S9A Fig**). By analysis of the 3D chromatin organization in embryos at high resolution, not only extended but also shorter domains were resolved. It was found that TADs in embryos cover 98% of the region under study on chromosome 2R [30], but depending on chromatin properties, can be divided into one active and three inactive types: repressed, PcG, and HP1. As much as 88% of the total length of rb-bands corresponds to inactive TADs. On the other hand, a substantial proportion of the length of an inactive TAD corresponds to rb-bands (**S9C and S9B Fig**).

The rb-bands are substantially depleted of TAD boundaries (**S9E Fig**); 53% of TAD boundaries fall within 10-kb intervals centered around each boundary of black bands (the expected value in the case of an independent distribution is 15%). This finding provides further support to the hypothesis that TAD boundaries are in fact the boundaries of black bands in polytene chromosomes.

We analyzed the patterns of distribution of proteins specific for the silent chromatin type as well as replication timing around the boundaries between different TAD types as identified elsewhere [30]. **S8 Fig** reveals that, as at the rb-band boundaries, there is a sharp increase in the expression of SUUR, LAMIN, H1, and H3K27me3 and a typical averaged replication profile at the boundary between active and repressed TADs. Similar to rb-bands, repressed TADs manifested a correlation between size and such measures as mean replication time, mean SUUR scores, and mean LAMIN scores (**S9F Fig**).

Consequently, it can be concluded that TADs correspond to rb-bands or groups of closely spaced rb-bands in polytene chromosomes.

### Distribution of SUUR and H3K27me3 in salivary gland polytene chromosomes verifies rb-band mapping

For the most part, our band-mapping approach depends on the data coming from cell lines. The best way to verify our mapping is by comparing the results with the distribution of the proteins in salivary gland polytene chromosomes. Good candidate markers of rb-bands are SUUR and H3K27me3 because they show a sharp change at band boundaries in a profile averaged for all such boundaries. Data on the distribution of SUUR and H3K27me3 in polytene chromosomes were published recently [61]. The resolution of this mapping is very high, allowing the behavior of these markers at the boundary of each band to be monitored on an individual basis (**S1 Fig**).

We visually assessed the correspondence between bands and zones with increased levels of these markers (for results, see **S1 Table**). Of 159 rb-bands, 131 showed exact coincidence (with a possible shift not exceeding 3 kb) between the bands and zones with high SUUR scores. For 19 bands, the high-SUUR-score zone in part coincided with a band. We believe that in these cases, the shifts of boundaries are due to the inaccuracy of our prediction. There were only six bands for which no local increase in SUUR was observed; it was found that all these bands are low on ruby chromatin. It is possible that the chromatin type in these bands is different from that in black bands.

The fraction of bands perfectly coinciding with the increased H3K27me3 score was somewhat lower than that of bands coinciding with an increased SUUR score: 82 out of 159. Among them, six are the bands perfectly coinciding with zones containing extremely high levels of H3K27me3. Domains such as these correspond to the domains that bind Polycomb protein [61].

In **S1 Table**, we singled out 52 bands that do not manifest either moderate or strong gene expression (not even on their boundaries) and show very good correspondence with local increases in SUUR and H3K27me3. The accuracy of our mapping for these bands is, by our estimate, up to 2 kb.

The question how the presence of a transcriptionally active gene at the band boundary influences the morphology of this boundary requires special research. Detailed cytogenetic analysis of small locus 61C7-61C8 on chromosome 3L by high-resolution microscopy has been reported in ref. [62]. Those authors assigned positions of four bands identified in this region to the four-color model of chromatin [4] and showed that in three cases (when the boundary of the band that was predicted by the model did not include lazurite chromatin and an actively transcribed gene), the model worked accurately. When lazurite chromatin was located on the boundary of a predicted band, the morphologically decompacted chromatin’s boundary shifted (Fig 4 in ref. [62]). Thirty-nine bands show moderate or strong gene expression on their boundaries. In such cases, the SUUR and H3K27me3 enrichment zones sometimes corresponded to the band we predicted, sometimes they did not. It appears that the question as to how to shift the band boundary when transcription is a factor should be addressed in each individual case and requires special consideration. We can therefore introduce a ±5- to 10-kb correction to the boundary mapping resolution, depending on the size of the marginal gene being transcribed.

Thus, the distribution of SUUR and H3K27me3 in polytene chromosomes is, in our opinion, a very good criterion for predicting rb-band boundaries; however, the resolution of this prediction can be affected by local transcription.

## Discussion

In this work, we used the four-state chromatin model, previously published data on the chromatin localization of proteins, and in situ hybridization of annotated genes and identified the locations of all black bands of polytene chromosome 2R on a genome map.

The special feature of the four-state chromatin model is the generalization of data obtained from four cell lines. This generalization resulted in identification of two chromatin types—aquamarine and ruby—which show stable properties in all these cell lines [19]. Starting our mapping effort with identification of domains that have ruby chromatin in them and that are flanked by aquamarine chromatin, we roughly divided the genome into constitutively active and constitutively inactive zones.

To take into account the tissue-specific features of chromatin organization in salivary gland polytene chromosomes, we analyzed the morphology of polytene chromosomes, an extensive pool of “experimental cytology” data, and data on gene expression in salivary glands and, whenever deemed necessary, introduced corrections into initial predictions. The results of our work reveal good performance of this approach.

Yet another unique feature of the four-state chromatin model is that it reveals the chromatin type (specifically, aquamarine) that is enriched with interband-specific proteins; no other models of clustering chromatin proteins can do this. According to the most recent high-resolution Hi-C data from embryos [28], TAD boundaries correspond with high resolution to polytene chromosome interbands, whereas black and gray bands are the visualization of topological domains with different types of DNA folding. Thus, the four-state chromatin model allows the boundaries of physical, not epigenetic domains to be found. The work by Hou and colleagues [29] clearly indicates that these boundaries are not always the same.

The choice of aquamarine chromatin as potential band boundaries is supported well by comparing our coordinates with the distribution of SUUR and H3K27me3 on polytene chromosomes: as we showed, these two are markers of black bands and display sharp changes on their boundaries. Thus, the approach used in this work and schematically detailed in **Fig 2**can in our opinion be conveniently used to identify the coordinates of specific polytene-chromosome black bands with high accuracy. For most black bands, accuracy of 2–10 kb is attainable.

With the band coordinates inferred, we performed a detailed comparison of replication profiles from diploid cells and replication patterns observed in polytene chromosomes and analyzed the properties of black bands.

We demonstrated a considerable degree of similarity in replication timing between salivary gland polytene chromosomes and diploid cells. In both object types, the zones between black bands correspond to early replication initiation zones. This result is consistent with the observation that most ORC2-binding sites are in aquamarine chromatin corresponding to interbands [4]. Rb-bands in different cell types have a U-shaped replication profile, which implies that replication in them proceeds from the boundaries to the center, leading to a local delay in replication completion, this delay being proportional to the band length.

The averaged boundary replication profiles in INTs that we built from previously published cell culture data [33] are consistent with the prediction that very few sites within the intervals between rb-bands initiate replication in each replication cycle. The typical size of replicons, 80 kb, originating from the early replication initiation zone (data from cell cultures) fits this model well too [33]. Analysis of stretched DNA fibers in *D*. *nasuta* polytene chromosomes [63] has revealed that the replicons initiated in the early S phase are each 64 μm in size on average, which should amount to more than 120 kb. The fact that the replicons are that long provides further support to the hypothesis that the replication origins fired during one cycle in a particular DNA molecule should be well spaced. While analyzing replication in partially denatured polytene chromosomes, researchers observed temporal and spatial asynchrony in replication initiation in parallel fibers and proposed that this asynchrony is one of the main reasons for continuous labeling in polytene chromosomes [63]. Although these data come from a *Drosophila* species irrelevant to us and the typical sizes and genomic distances may be different to some extent [64], we propose that the organization of replication in polytene chromosomes is conserved across *Drosophila* species [65].

**Fig 8:**
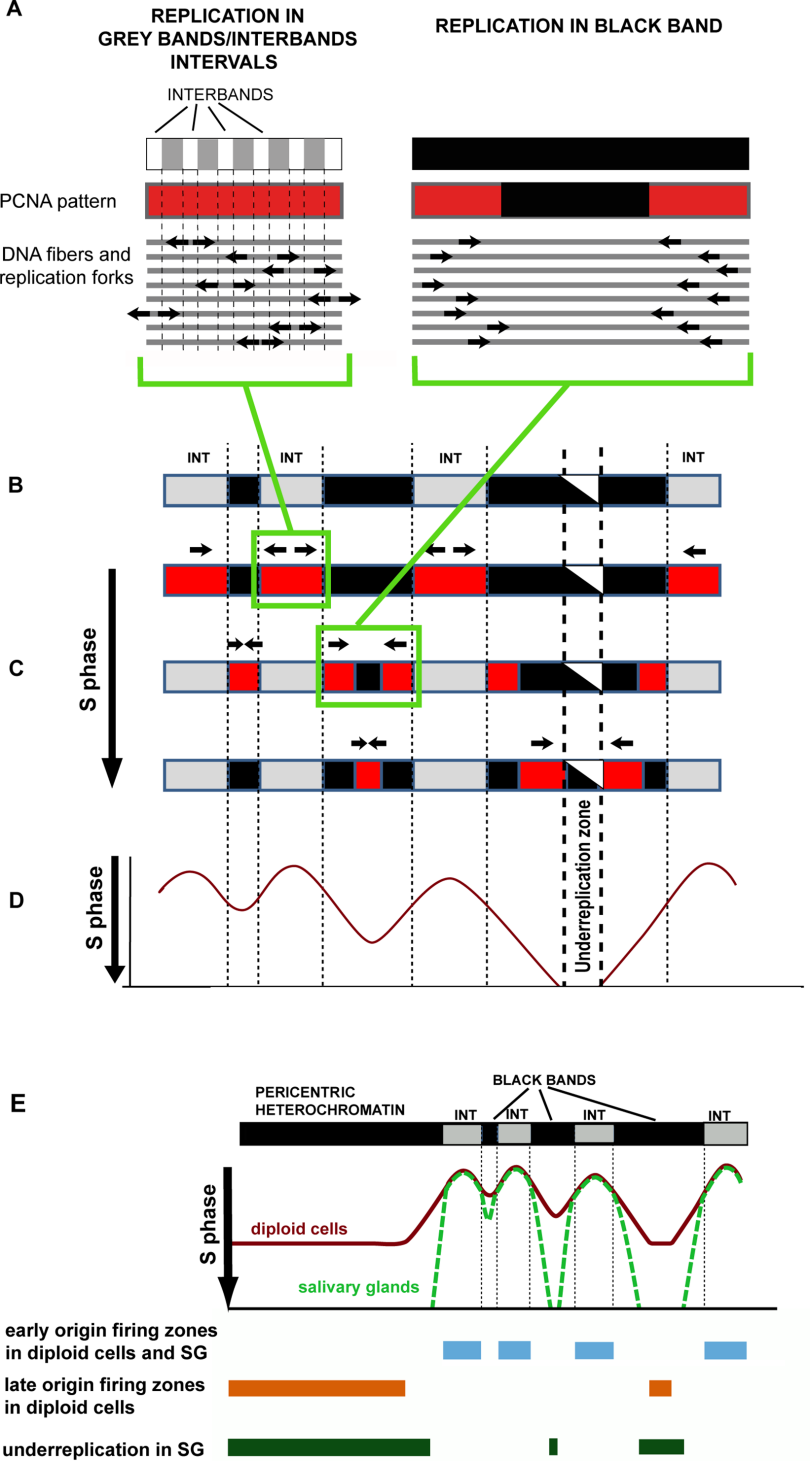
The logic of changing patterns in polytene chromosomes. **Links between these patterns and events at the DNA sequence level. (A)** A schematic of replication fork locations and the corresponding PCNA patterns in the zone of alternating grey bands and interbands and in an rb-band (black in the figure). **(B)** A schematic of a polytene chromosome region with three rb-bands (black in the figure) of different lengths. Intervals between rb-bands appear as grey bands and interbands. **(C)** Consecutive changes in PCNA binding patterns (red in the figure) during the S phase. Top to bottom: continuous labeling substage; the substage at which the label is seen in all rb-bands; the late S phase, when only the thickest bands get labeled. The central part of the thickest band contains a region that never undergoes replication. **(D)** Replication fork locations depending on time (time passes in the downward direction). Replication fork locations were inferred under the assumption that replication is initiated randomly at each INT point once per INT in each replication cycle, with little asynchrony. Differences in replication rates between different genomic regions were ignored. The portion of the profile corresponding to the under-replication zone is beyond the S phase. If the differences in replication rates between rb-bands were to be considered, the local minima would be deeper. **(E)** A model reflecting similarities and differences in replication timing between diploid cell chromosomes and salivary gland polytene chromosomes. Averaged replication profiles are identical in early replication initiation zones. A portion of the genome in salivary gland polytene chromosomes is slower to replicate because of slow replication in “black” bands and the absence of late origin firing; this portion stays under-replicated.

A schematic derived from original and previously published data is presented in **Fig 8**. The schematic illustrates how replication patterns change in polytene chromosomes and how these patterns are linked to events in DNA sequences. At the beginning of the S phase, replication is initiated in INTs, which may contain a large number of potential replication initiation sites. In each DNA strand, an initiation event occurs only once per INT in a random interband. Initiation events in different interbands can occur in asynchrony, either in different INTs or in the same INT on the parallel DNA strands of a polytene chromosome. Replication forks move through INTs in the opposite directions from the site of replication initiation and eventually enter the nearest rb-bands (**Fig 8A**). After all INTs have completed replication, the replication fork should be detectable only in rb-bands. This situation is consistent with the observed inverted PCNA pattern, when all black bands produce the signal that the intervals do not.

By analysis of stretched DNA fibers in *D*. *nasuta* polytene chromosomes, it has been demonstrated that the rates of replication fork movement in polytene chromosomes during the late S phase are on average one-tenth of those in the early S phase [63]. In our opinion, upon entering polytene chromosome rb-bands, replication forks slow down. That is why, although some rb-bands are shorter than the flanking intervals, we can observe all “black” bands undergoing replication at once. Replication in these bands goes on until the forks moving toward each other meet. In the longest rb-bands, forks fail to meet before the end of the S phase, leading to under-replication (**Fig 8B–8D**). In *D*. *melanogaster*, replication rates depend on SUUR [51, 66]. We demonstrated that all rb-bands are enriched with SUUR both in salivary gland polytene chromosomes and in diploid cells. According to [67], local artificial tethering of SUUR to an early replicating region of a salivary gland polytene chromosome causes delayed replication there. It can be proposed that this protein plays an important role in delayed replication associated with all rb-bands genome-wide.

Evidence exists that the S phase of the endocycle is quite different from that in diploid cells. The former is distinguished by under-replication of a large part of the genome and low expression of genes involved in replication. The presence of the intra-S checkpoint in salivary gland cells is questionable, and so is activation of any late-firing origins [51, 68–70]. Nevertheless, our results reveal a substantial similarity in replication timing for the euchromatic arm of the whole chromosome. What underlies this similarity is in our opinion the organization of the *Drosophila* genome. The genome consists of alternations of domains capable of initiating early replication (INTs) and domains with the potential to initiate replication late in the S phase. These late domains vary in size, but seldom are they longer than a few hundred kilobases. In diploid cells, these relatively short domains are replicated by replication forks coming from border origins of replication and complete replication before the classic late S phase, which is when late-firing origins activate. Thus, replication of a large portion of a euchromatic arm is, in the classic sense, early replication. Only the most extended bands and regions of pericentric heterochromatin initiate replication in the late S phase (**Fig 8E**). The question of whether replication initiation events occur in the bands is not easy to answer. Schwaiger and coworkers [33] have analyzed replication profiles and concluded that extended late-replication zones in cell cultures contain origins initiating replication shortly before the end of the S phase. It can be assumed that the replication origins located in rb-bands do not bind all proteins required for independent initiation of replication, and replication on these origins cannot be initiated before a fork comes from outside; these properties are typical of regions showing a U-shaped replication profile in mammals [54, 71]. The same is suggested by recent studies of the genome-wide distribution of the Mcm2-7 helicase complex in *D*. *melanogaster* [72].

Multiple published comparisons of replication profiles for different tissues of the same organism suggest that each cell type has its own schedule of origin activation. One study on individual IH regions indicates that all the 60 analyzed regions are late replicating in cultured cells [17], but inside those regions, there are local zones of early replication. After artificial induction of transgene expression in IH, there are also local changes in replication timing [73].

In cell cultures, early-replication zones within rb-bands can be identified by analysis of the outliers in the boxplots of the averaged boundary replication profile (**S5 Fig**). Among all the rb-bands, only two had early-replication zones spanning them from end to end. It can be theorized that in different tissues, most bands similar in size undergo replication within a similar time interval in the S phase, and gene activation in these bands makes the corresponding fragment of the band earlier replicating.

It can be concluded that the alternation of rb-bands and INTs forms the basis of the pattern of replication timing in *D*. *melanogaster*. This organization is conserved in eukaryotes. It has been demonstrated that a substantial portion of the mammalian genome represents the alternation of replication initiation zones, in which early master origins lie, and U-shaped replication zones, in which initiation occurs at virtually random positions and in a cascadelike manner, shaping the profile accordingly [54, 74–77]. The initiation zones are notable for active transcription and high gene density. The boundaries of these zones correspond to those of topological domains [74–77].

IH regions represent a separate fraction of black bands, grossly corresponding to the most extended and late-replicating bands (group LR5). We demonstrated that all rb-bands, including small ones, share a large number of properties with IH regions. This is direct evidence that among all genomic regions, IH regions do not stand out as some special type of sequences. Genes in any rb-band tend to be expressed in a limited number of tissues and, according to GO analysis, these regions are enriched with tissue-specific genes. By contrast, the intervals between black bands are enriched with genes that are highly expressed in most tissues chosen for analysis here. Each rb-band appears as a combination of repressed chromatin types; however, open chromatin can be found in its boundary regions, pointing to a similarity between bands and TADs [29]. We confirmed that the boundaries of black bands correspond to those of topologically associating domain or sub-domains. TADs represent a stable level of genome organization during development both in mammals and in *Drosophila*. It has been demonstrated that the partitioning of genomes into physical domains correlates with gene density and transcription distribution [78–80]. These features are closely associated with replication timing [16, 78–80]. That late replication correlates with LADs has been demonstrated in both *Drosophila* and mammals [81, 82].

The results of our work suggest that *Drosophila* polytene chromosomes can serve as vivid visualization of the organization of the eukaryotic genome, which is conserved between *Drosophila* and mammals. The characteristic pattern of polytene chromosomes—the compacted black bands alternating with less compact grey bands and interbands—reflects the partitioning of the *Drosophila* genome into domains with contrasting properties.

## Materials and methods

### Fly stocks and analysis of polytene chromosome morphology

Flies were raised on standard *Drosophila* cornmeal–yeast–agar medium at 18°C and 25°C. The Oregon R stock from the Bloomington Drosophila Stock Center was used as wild-type control. The *SuUR*^*ES*^ mutation was first described by Belyaeva and the co-workers [83].

For acetic orcein staining of polytene chromosomes, glands were dissected in PBS (137 mM NaCl, 3 mM KCl, 8 mM NaH_2_PO_4_ and 2 mM KH_2_PO_4_), transferred to aceto-orcein solution (1% orcein in 45% acetic acid) for 10–15 min then to 55% lactic acid for 1–2 min and squashed. Phase-contrast images were captured with an Olympus BX51 microscope using a DP52 camera with a 100× oil immersion objective lens.

### Genome mapping of black bands

A detailed description of mapping is provided in **S1** Text.

We relied on the detailed cytological map of chromosome 2R [47] and an electron microscopic map by Saura and the co-workers [45, 46]. All maps are available in FlyBase (https://wiki.flybase.org/wiki/FlyBase:Maps). Polytene chromosome morphology was analyzed using previously published electron microscopic photographs of separate regions of polytene chromosome 2R [45, 46, 84–86], and aceto-orcein preparations.

All the genomic coordinates used in this work are provided in *D*. *melanogaster* Release 5 assembly. The UCSC Genome Browser [87] was used for data visualization. The genomic coordinates of the bands were identified using the coordinates of four chromatin types from a work by Zhimulev and the co-workers [4] and the coordinates of nine modENCODE chromatin types from a work by Kharchenko and the co-workers [22]. CHRIZ enrichment peaks in the chromosomes of the cell lines (modENCODE_275, modENCODE_277, modENCODE_278, and modENCODE_276) were downloaded from <http://modencode.org/>. The list of genes with in situ hybridization data published for them was extracted from FlyBase [44] using the Batch Down tool. The FlyBase coordinates of the bands were downloaded using the UCSC Table Browser [88]. For band mapping, we used salivary gland gene expression data available from FlyAtlas [89], as well as expression data generated by modEncode [90] (data freeze March 2016). Gene coordinates were downloaded from FlyBase [44] release 5 version 57. To extract all data on the physical mapping of genes in chromosome 2R from FlyBase, we used the Batch Download function in the Experimentally Determined Cytological Location section.

We determined the naming accuracy for each band against Bridges’ map [47] by how many times data on experimental mapping of genes onto that particular band and its adjacent regions in the polytene chromosome have been referenced in independent sources (**S1 Table**).

To verify the mapping of large bands, we used 186 photographs (the most relevant from among 2255 kindly provided by Todd Laverty) resulting from *in situ* hybridization of P inserts to chromosome 2R from the BDGP project [49]. The names of inserts were used as queries for retrieving the genomic coordinates of the inserts or the names of their target genes from FlyBase.

### Mapping replication in salivary gland polytene chromosomes

To detect replication events in salivary gland polytene chromosomes, we immunostained squashes of Oregon R and *SuUR*^*ES*^ wandering third instar larval polytene chromosomes with antibodies to PCNA. Salivary glands were dissected in Ephrussi-Beadle solution (7.5 g NaCl, 0.35 g KCl, 0.21 g CaCl_2_ per liter) supplemented with 0.1% Tween-20. Glands were then transferred into a formaldehyde-based fixative (0.1 M NaCl, 2 mM KCl, 10 mM NaH_2_PO_4_, 2% NP-40, 2% formaldehyde) for 1 min. Next, the salivary glands were placed in an acetic acid–formaldehyde mix (45% acetic acid, 3.2% formaldehyde) for 1 min and squashed in 45% acetic acid. Squashes were snap-frozen in liquid nitrogen; the coverslips were removed. Slides were incubated in 70% ethanol for 5 min twice and stored in 70% ethanol at −20°C. Slides were washed three times in PBST (137 mM NaCl, 3 mM KCl, 8 mM NaH_2_PO_4_ and 2 mM KH_2_PO_4_; 0.1% Triton X-100 or Tween-20) for 5 min. The primary mouse anti-PCNA (PC10, Abcam, ab29, 1:500) antibody were added in a blocking solution (0.1% BSA in PBST) and incubated in a humid chamber for 2 h at room temperature. The squashes were washed three times for 5 min in PBST, and the secondary Texas Red-labeled goat anti-mouse IgG specific conjugates (ab6787, Abcam, 1:200) in blocking solution were applied. Incubation lasted for 1 h and was followed by washing in PBST three times for 5 min each. DNA was visualized by DAPI staining (0.1 μg/ml in PBST, D1306, Invitrogen) for 5 min.

The total number of preparations analyzed was 35 (more than 1000 nuclei) for Oregon R and 12 (more than 500 nuclei) for *SuUR*^*ES*^. All whole 2R chromosomes were analyzed on the preparations and to each chromosome a particular staining pattern (that is, a set of replicating bands) was assigned. All these patterns were classified into six designated ER and LR1 through LR5. To each rb-band, the substage at which its replication ends was assigned. Although the order of band replication is overall consistent, there was a slight variability to it. That is why substaging assignments were finalized after all preparations were analyzed.

It was previously demonstrated that IH bands complete replication earlier in *SuUR*^*ES*^ mutants than in the wild-type flies. As a result, late S phase reveals replication patterns that suggest replication only in the chromocenter and few bands, which is untypical of the wild type [38]. At the same time, the sets of patterns typical of either early or mid-S phase are virtually the same; however, the staining pattern and structure of bands in *SuUR*^*ES*^ is clearer than in the wild type, because underreplication is not there [39]. The figures provide LR2-LR5 data for the wild-type line. For earlier substages, *SuUR*^*ES*^ was used.

### Data analysis

Data processing, plotting and visualization were achieved using statistical language R [91] and Microsoft Excel.

Replication timing data for diploid Kc and Cl8 cells [33] were downloaded from ReplicationDomain at http://www.replicationdomain.com/. DamID data were retrieved from GEO: LAMIN (GSE20313) in Kc cells [60]; SUUR (GSE22069) in Kc cells [21]; and SUUR (GSE33873) in salivary glands [59]. The coordinates of physical domains were taken from supplementary materials to works by [26, 27, 29, 30]. Hi-C data for salivary glands [26] were analyzed using accession GSE72512 in GEO.

Analysis of the averaged distribution of replication timing scores, protein abundance and ratios of chromatin types around the boundaries of rb-bands included all rb-band/INT boundaries except those that were closer than 10 kb apart. Data on the distribution of the values of each feature were aligned relative to the boundaries so that the within-boundary area was on the right and the off-boundary area was on the left. Next we analyzed the distribution in both directions within fixed 1-kb or 10-kb windows.

When constructing plots with replication score data normalized to median length of rb-bands **(Fig 5J and 5K)**, each rb-band and each INT was divided into 20 bins. The medium replication score was determined within each of the bins. With these data, a separate smoothened line was drawn separately for each band group (LR0-1, LR2-3, LR4-5) and INTs by LOESS regression with R.

The percentage of each of the different chromatin types in rb-bands and INTs was determined using Galaxy [92] using the *Intersect* tool in the *Operate on Genomic Intervals* section.

Gene expression in bands and INTs was analyzed using Expression data generated by modENCODE [43, 90] (data freeze March 2016). Gene coordinates were downloaded from FlyBase [44] release 5 version 57. Only loci that fully overlapped with an rb-band or an INT counted. The list of the testis-specific genes was borrowed from a paper by Shevelyev and the co-workers [55].

The enrichment of GO-annotated genes [56] in the bands and INTs was analyzed using the GOrilla tool [93] with the following settings: the running mode: two unranked lists of genes (target and background lists); target genes: genes annotated as protein-coding genes and fully included in rb-bands or INTs. The background set was all protein-coding genes in the genomic region chr2R:1670600–21140601 corresponding to our study region of chromosome 2R.

## Supporting information

S1 Fig Salivary-gland data help predict and refine the positions and boundaries of rb-bands. Data found in various sources regarding salivary gland polytene chromosomes are put together and presented for entire chromosome 2R. Top to bottom: **Experimental cytology:** FlyBase-referenced genes having experimental mapping data. **H3K27me3, SG:** a H3K27me3 ChIP-seq profile in salivary glands. Data are presented as quantile normalized log2(IP/inp) values [48]. **SUUR, SG:** a SUUR DamID profile in salivary glands. Data are presented as log10(P) units, where P is the significance level assessed with Fisher’s exact test [48]. **Replication timing:** grey-shaded rb-bands, from latest (black) to earliest (light-grey) replicating bands. **Hi-C SG:** a normalized Hi-C interaction map (15-kb bins) from salivary gland cells [26]. **Expression, SG:** color-coded gene expression levels (according to modENCODE [43, 90]): grey, very low and low; green, moderate; orange, high; red, very high.
(TIF)

S2 Fig Our mapping of black bands in polytene chromosomes versus data on *in situ* hybridization of P inserts from the BDGP project [49]. **(A–C)** Region 51F-52C of chromosome 2R. This region is difficult to map because it contains a large number of closely spaced morphologically similar bands. **(D–F)** Region 60A–60F of chromosome 2R. **(A)** In the photographs: results of *in situ* hybridization of probes corresponding to the P element in the following lines: 1) l(2)k11320, 2) l(2)k06203, 3) l(2)k03617, 4) l(2)k16004, 5) l(2)14707, 6) l(2)k08015, 7) l(2)k06403, 8) l(2)k04218, 9) l(2)k17029, 10) l(2)k03308, and 11) l(2)k07201. Each photograph is linked to the region on the genome map in which the insert was located. On the map: rb-bands and chromatin types according to the 4-state model. **(B)** A part of Bridges’ detailed map of chromosome 2R (regions 50D–52C) [47]. **(C)** A part of Bridges’ detailed map of regions 50D–52C (B). Blue normal and T-headed arrows indicate hybridization loci according to our mapping prediction. Red normal and T-headed arrows indicate their locations according to the photographs (A). **(D)** In the photographs: results of *in situ* hybridization of probes corresponding to the P element in the following lines: 1) l(2)k13108 site2, 2) l(2)k11035, 3)l(2)k07623, 4) l(2)k04201, 5) l(2)k04405 site1, 6) l(2)k01208, 7) l(2)01296, 8) l(2)k12101, 9) l(2)k05318, 10) l(2)03267, 11) l(2)k00808, 12) l(2)04012, 13) l(2)k10502, 14) l(2)k04809, 15) l(2)k03704, 16) l(2)k15609, 17) l(2)10481, and 18) l(2)k05826. Each photograph is linked to the region on the genome map in which the insert was located. On the map: rb-bands and chromatin types according to the 4-state model. **(E, F)** A part of Bridges’ detailed map of regions 60A–60F [47]. Designations as in C. Although the resolution of *in situ* hybridization does not always allow the probe to be located accurately to a thin band, all the photographs are fully consistent with our mapping.
(TIF)

S3 Fig Band replication substaging in polytene chromosome 2R. Top to bottom: six S-phase substages designated as ER (early replication) and LR0 to LR5 (late replication). Because this substaging is somewhat arbitrary and because there is variation in replication observability in separate bands at each substage, bands were assigned to substages following analysis of a large number of preparations. For this reason, the names of some substages end with more than one number. **(A)** Region 42-46 of chromosome 2R. **(B)** Regions 46-50. **(C)** Regions 50–56. **(D)** Regions 56-60.
(PDF)

S4 Fig The rb-bands are depleted for replication initiation sites. **(A)** Density of ORC2 peaks (peaks/100 kb) (according to [51]) in Bg3 cells. **(B, C)** Density of replication origins (peaks/100 kb) identified in Bg3 (B) and S2 (C) cells by next-generation sequencing of small nascent leading strands [53].
(TIF)

S5 Fig Zones of changes in replication timing normally correspond to band fragments, not to whole bands. Boxplots of replication time scores in Kc **(A)** and Cl8 **(B)** cells for all sequences within 10-kb windows at the same position relative to the band boundary (shown as a red line). Right boundary data were mirrored and combined with left boundary data. Within-band regions are to the right of the border; outside regions are to the left. On the x-axis: distances from the border. On the y-axis: replication time scores (data were taken from [33]), with +6 corresponding to early replication, and −5 to late replication.
On the right: all bands containing DNA sequences with replication scores corresponding to the outliers in the boxplots. For each band, the presence in a group from LR0 to LR5, the number of the windows with replication times in the outliers, and the size are indicated. To be noted most outliers correspond to the windows found at rb-band/INT borders. In Kc cells, an extended (~80 kb) outlier zone is observed in band 47D1-6 (windows 2 through 10; the total length being 250 kb). Of note, 47D1-6 is not a solid band, but a cytologically complex region, in which our interband criteria failed to identify individual bands. Extended outlier zones are observed in bands 49A1-2 (50 kb, or more than 60% of band size) and 50A1-4 (50 kb, or more than 30% of band size). In C18 cells, extended outlier zones are observed in bands 45C1-2 and 45E1-2. Here the zones span the bands and continue beyond them. In all other bands, the outlier zones are normally not longer than 20 kb each. It can be concluded that zones of changes in replication timing normally correspond to band fragments, not to entire bands.
(TIF)

S6 Fig Distribution of different chromatin types in size-ranked rb-bands and INTs. The rb-bands and INTs are presented at the same scale and are size-ranked. The scale (kb) is indicated below. **(A)** Distribution of four chromatin types [4]. **(B)** Distribution of five chromatin types in Kc cells [21]. **(C)** Distribution of differently compacted chromatin types in S2 cells (after [58]). **(D)** Distribution of four TAD types (after [30]).
(TIF)

S7 Fig Distribution of different chromatin types at the boundaries between rb-bands and INTs. **(A)** An averaged distribution of four chromatin types (after [4]) at locations close to the boundaries of LR0-1, LR2-3, and LR4-5 rb-bands. **(B)** Averaged distribution of five chromatin types in Kc cells (after [21]) at locations close to the boundaries of LR0–1, LR2–3, and LR4–5 rb-bands. **(C)** An averaged distribution of differently compacted chromatin types in S2 cells [58] at locations close to the boundaries of LR0–1, LR2–3, and LR4-5 rb-bands. The band boundary is at position zero. To the right: band material; to the left: no-band material. The increment is 10 kb. Numbers of boundaries analyzed: for LR0-1/INT n = 113, for LR2-3/INT n = 76, and for LR4-5/INT n = 36.
(TIF)

S8 Fig Similarity in the distribution of repressed chromatin markers at the boundaries between rb-bands and INTs, E and D domains, and active and repressed TADs. Averaged profiles of the distribution of scores for repressed chromatin markers close to the boundaries between rb-bands and INTs, D- and E-domains (E domains less than 100 kb were removed), TADs of different types (null/active, Pc/active, null/Pc), and TADs of the same type (null/null, active/active). Right-boundary data were mirrored and combined with left boundary data. Markers: SUUR in salivary glands (data were taken from [59]), SUUR in Kc cells data were taken from [21]), LAMIN in Kc cells (data were taken from [60]), histone H1 in Kc cells (data were taken from [21], and H3K27Me3 in primary spermatocytes (after [31]). Boundaries that were closer than 10 kb apart were removed from the analysis. Numbers of boundaries analyzed: for LR0-1/INT n = 113, for LR2-3/INT n = 76, for LR4-5/INT n = 36. For D/E > 100 kb boundaries n = 832. For TAD/TAD boundaries: Active/Null n = 246, Active/Pg n = 62, Null/Pg n = 49, Active/Active n = 174, Null/Null n = 189.
(TIF)

S9 Fig The rb-bands correspond to TADs. **(A)** Hi-C data from different sources on the region 45F-46B of chromosome arm 2R (approximately 2 Mb). Top to bottom: rb-bands; a normalized Hi-C interaction map (15-kb bins) from salivary gland cells [26]; TADs in salivary gland cells [26]; TADs in embryonic cells according to [30]: null type (purple), PcG type (blue), active type (red); TADs in Kc167 cultured cells [29]; Hi-C interaction maps (20-kb bins) of the four cell lines [27]. **(B)** Chromatin packaging in all rb-bands corresponds to chromatin packaging in TADs. Boxplots of contact probabilities inside bins along the diagonal on the normalized Hi-C interaction map published by Eagen et al. [26] for salivary gland cells. TADs are significantly different from interTADs (inTAD in the figure; ***p < E-21); all rb-bands are significantly different from INTs (***p < E-16; **p < E-3; Mann–Whitney *U* test). **(C)** The ratio of the overall lengths of genomic intervals corresponding to LR0–LR5 bands (black) and INTs (grey) in TADs of different types. In all inactive TAD types, the prevalent chromatin is the one corresponding to bands. **(D)** The proportion ratio of chromatin types corresponding to TADs of different types according to [30], in LR0-LR5 bands and intervals between them. **(E)** Rb-bands are depleted of TAD boundaries in embryos. The observed and the expected number (assuming a normal distribution along the chromosome) of TAD boundaries (after [30]) within rb-bands and at their boundaries. **(F)** Coefficients of correlation (with p values as indicated) between fragment size and such measures as replication timing in Kc and Cl8 cells, SUUR binding in Kc and salivary gland cells, and Lamin binding in Kc cells.
(TIF)

S10 Fig The Hi-C interaction map from polytene salivary gland cells, published by Eagen et al. [26] reflects the morphological features of particular regions in salivary gland polytene chromosomes. Top to bottom: polytene chromosome morphology (^#^according to Bridges, 1939 [47]; *an electron micrograph according to [45]; ^$^aceto-orcein staining, phase-contrast); the heatmap of Hi-C data on salivary glands [26]; TADs in salivary glands [26]; rb-bands; gene expression levels (according to modENCODE [43]). **(A)** Each pair of closely spaced bands, 57B2-3/57B4-6 and 56A1-2/56B1-2, forms a single TAD. The internal interband manifests a very special feature on the heatmap: within the triangles with nearly evenly distributed interactions, the interband bin shows strong interactions between internal sequences and weak interactions between external. **(B)** In region 46F, polytene chromosome morphology is variable: what is seen to the left of the large and always prominent band 47A1-2 is sometimes a group of distinct black bands and sometimes a series of decompacted grey bands. This variability is probably associated with the activity of the Hr3 gene (the orange rectangle on the expression map), which, according to modENCODE, starts to be highly expressed shortly before the prepupal stage. The heatmap interval corresponding to this variable region showed alternations of bins with different properties and looked contrasting against extended regions with relatively uniform patterns of interactions.
(TIF)

S1 Table Mapping of rb-bands on chromosome 2R. (XLS)

S2 Table The rb-bands extend the list of the previously published late-replicated bands. (XLS)

S3 Table GO terms significantly enriched in rb-bands and intervals between them. (XLS)

S4 Table Overlaps of rb-bands with LADs in Kc cells and TADs in salivary glands. (XLS)

S1 Text The strategy for mapping Rb-bands and examples of discordant cases. (PDF)
