# Supplementary figures and images for: Similarity in replication timing between polytene and diploid cells is associated with the organization of the Drosophila genome
Source: PLoS One. 2018 Apr 16;13(4):e0195207. doi: 10.1371/journal.pone.0195207 (PMC5902040; doi:10.1371/journal.pone.0195207)

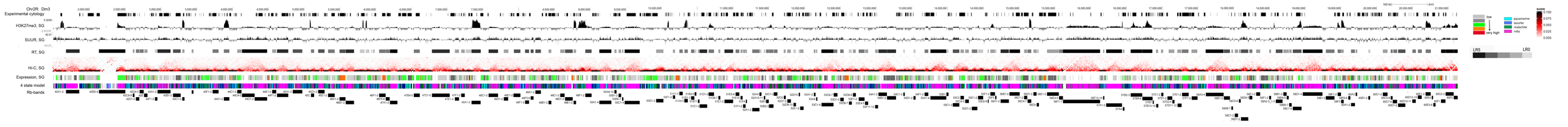

Supplement: S1 Fig — Data found in various sources regarding salivary gland polytene chromosomes are put together and presented for entire chromosome 2R. Top to bottom: Experimental cytology: FlyBase-referenced genes having experimental mapping data. H3K27me3, SG: a H3K27me3 ChIP-seq profile in salivary glands. Data are presented as quantile normalized log2(IP/inp) values [48]. SUUR, SG: a SUUR DamID profile in salivary glands. Data are presented as log10(P) units, where P is the significance level assessed with Fisher’s exact test [48]. Replication timing: grey-shaded rb-bands, from latest (black) to earliest (light-grey) replicating bands. Hi-C SG: a normalized Hi-C interaction map (15-kb bins) from salivary gland cells [26]. Expression, SG: color-coded gene expression levels (according to modENCODE [43, 90]): grey, very low and low; green, moderate; orange, high; red, very high. (TIF) [file pone.0195207.s001.tif]

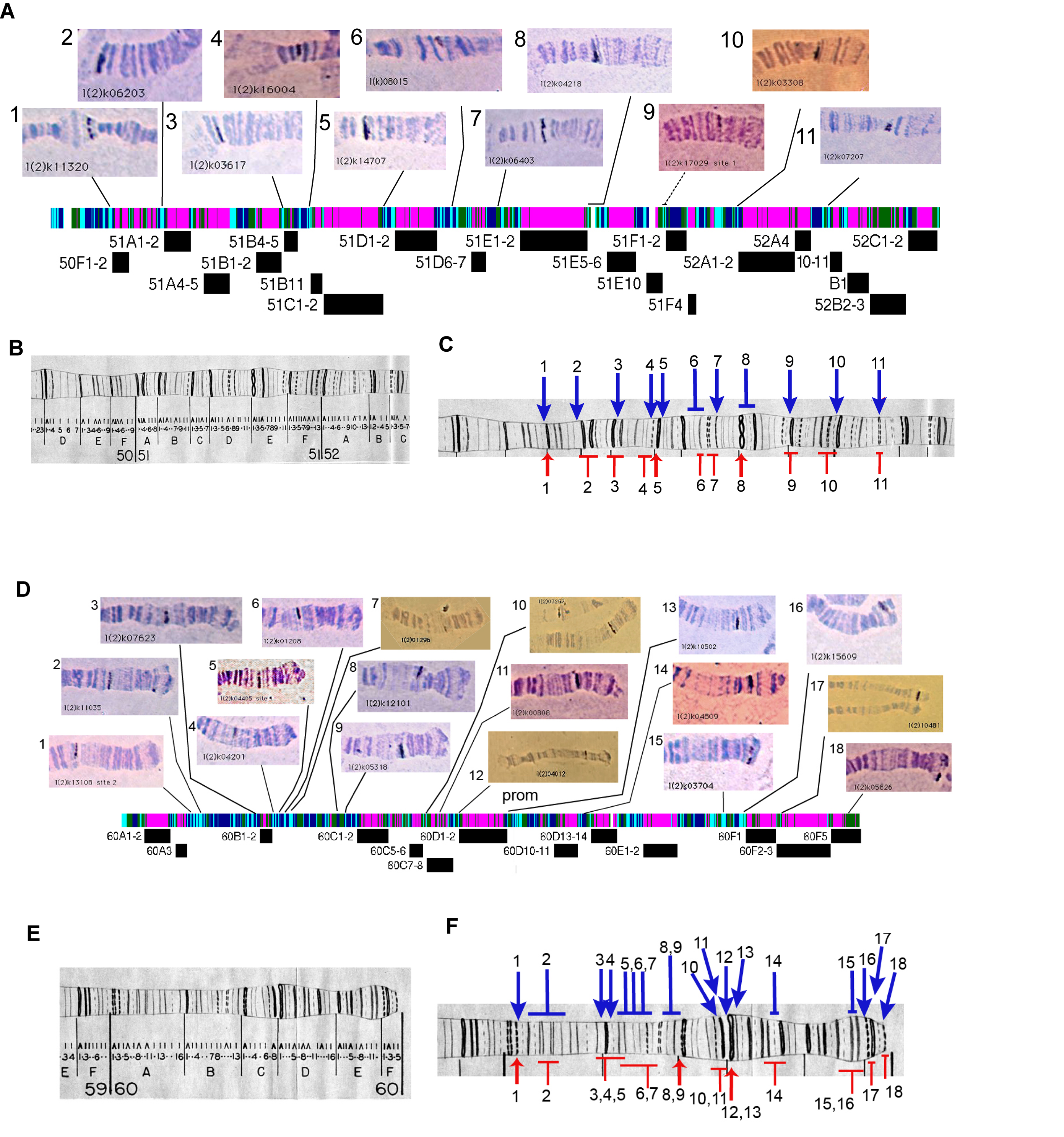

Supplement: S2 Fig — (A–C) Region 51F-52C of chromosome 2R. This region is difficult to map because it contains a large number of closely spaced morphologically similar bands. (D–F) Region 60A–60F of chromosome 2R. (A) In the photographs: results of in situ hybridization of probes corresponding to the P element in the following lines: 1) l(2)k11320, 2) l(2)k06203, 3) l(2)k03617, 4) l(2)k16004, 5) l(2)14707, 6) l(2)k08015, 7) l(2)k06403, 8) l(2)k04218, 9) l(2)k17029, 10) l(2)k03308, and 11) l(2)k07201. Each photograph is linked to the region on the genome map in which the insert was located. On the map: rb-bands and chromatin types according to the 4-state model. (B) A part of Bridges’ detailed map of chromosome 2R (regions 50D–52C) [47]. (C) A part of Bridges’ detailed map of regions 50D–52C (B). Blue normal and T-headed arrows indicate hybridization loci according to our mapping prediction. Red normal and T-headed arrows indicate their locations according to the photographs (A). (D) In the photographs: results of in situ hybridization of probes corresponding to the P element in the following lines: 1) l(2)k13108 site2, 2) l(2)k11035, 3)l(2)k07623, 4) l(2)k04201, 5) l(2)k04405 site1, 6) l(2)k01208, 7) l(2)01296, 8) l(2)k12101, 9) l(2)k05318, 10) l(2)03267, 11) l(2)k00808, 12) l(2)04012, 13) l(2)k10502, 14) l(2)k04809, 15) l(2)k03704, 16) l(2)k15609, 17) l(2)10481, and 18) l(2)k05826. Each photograph is linked to the region on the genome map in which the insert was located. On the map: rb-bands and chromatin types according to the 4-state model. (E, F) A part of Bridges’ detailed map of regions 60A–60F [47]. Designations as in C. Although the resolution of in situ hybridization does not always allow the probe to be located accurately to a thin band, all the photographs are fully consistent with our mapping. (TIF) [file pone.0195207.s002.tif]

A

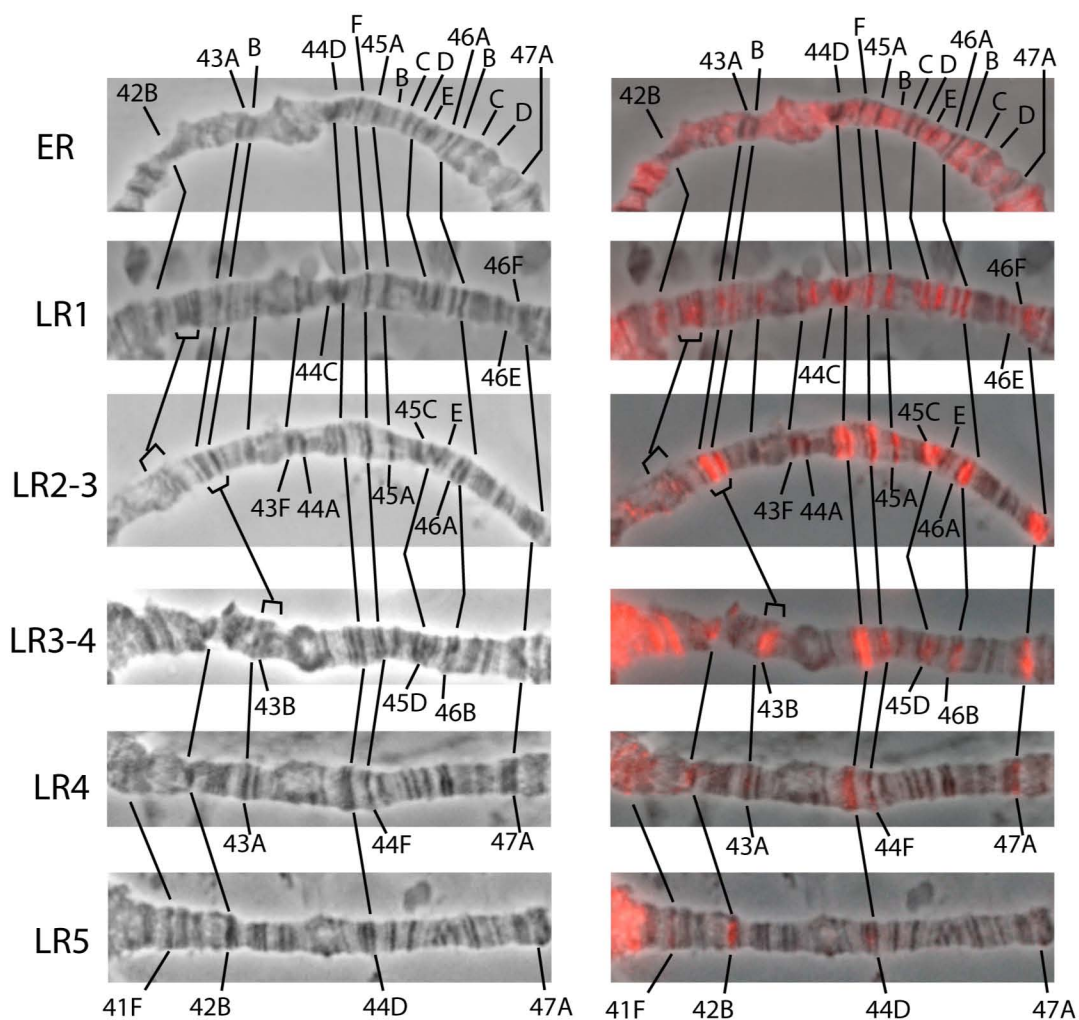

B

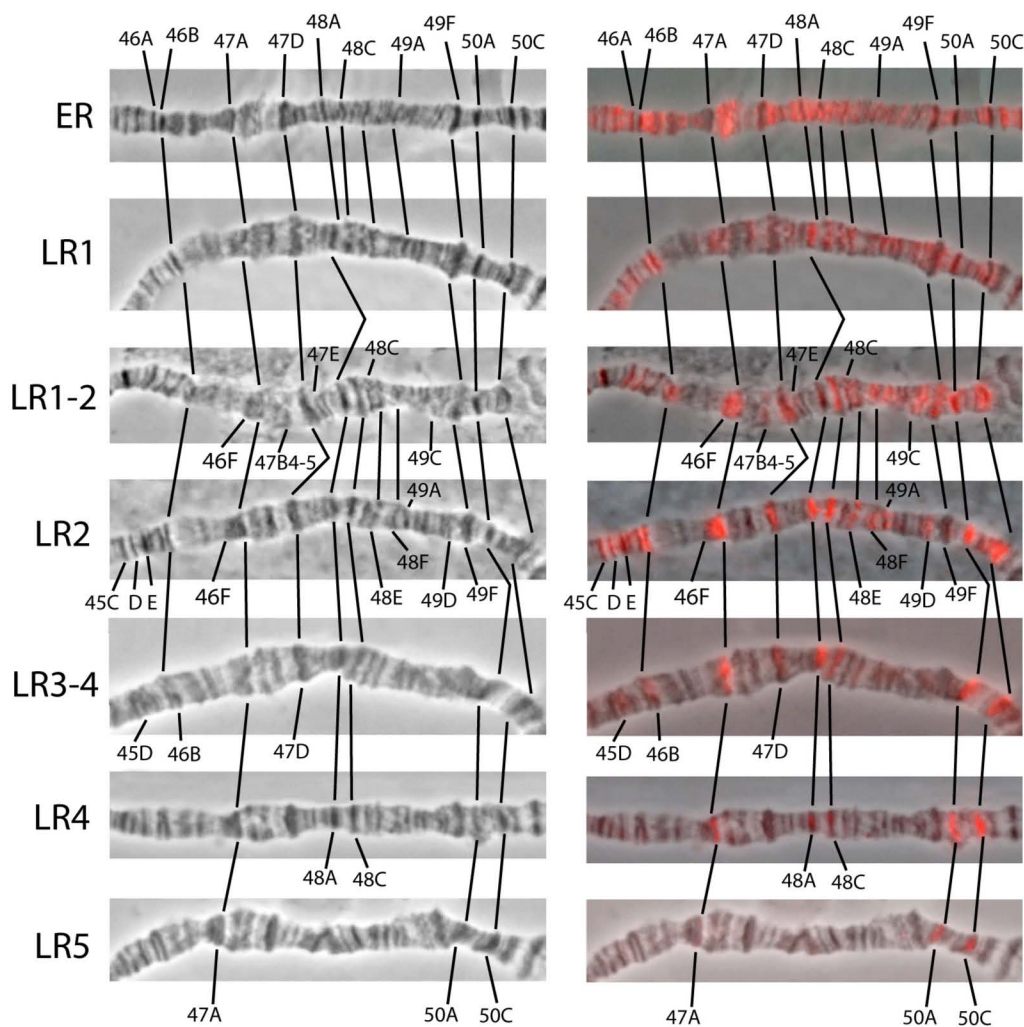

C

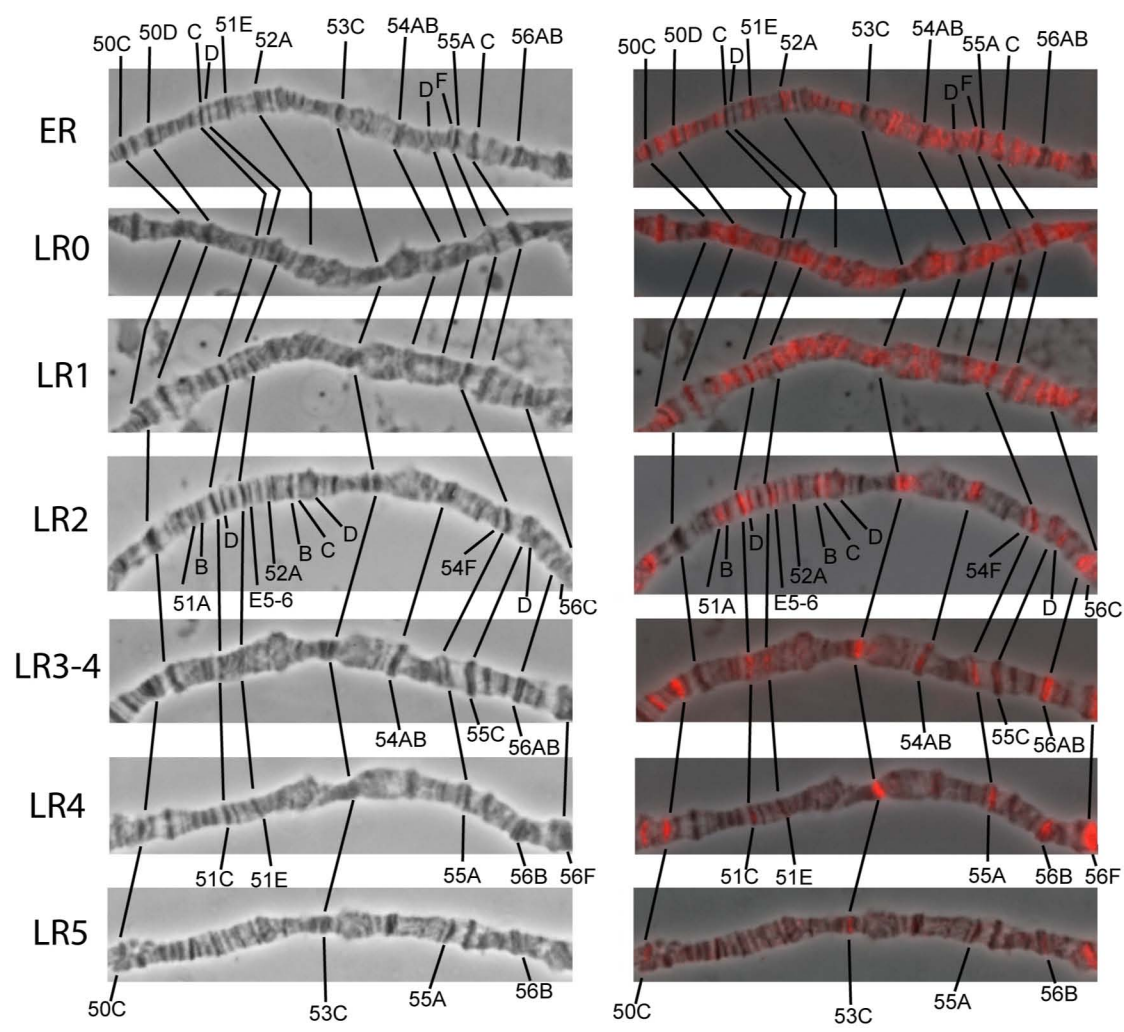

D

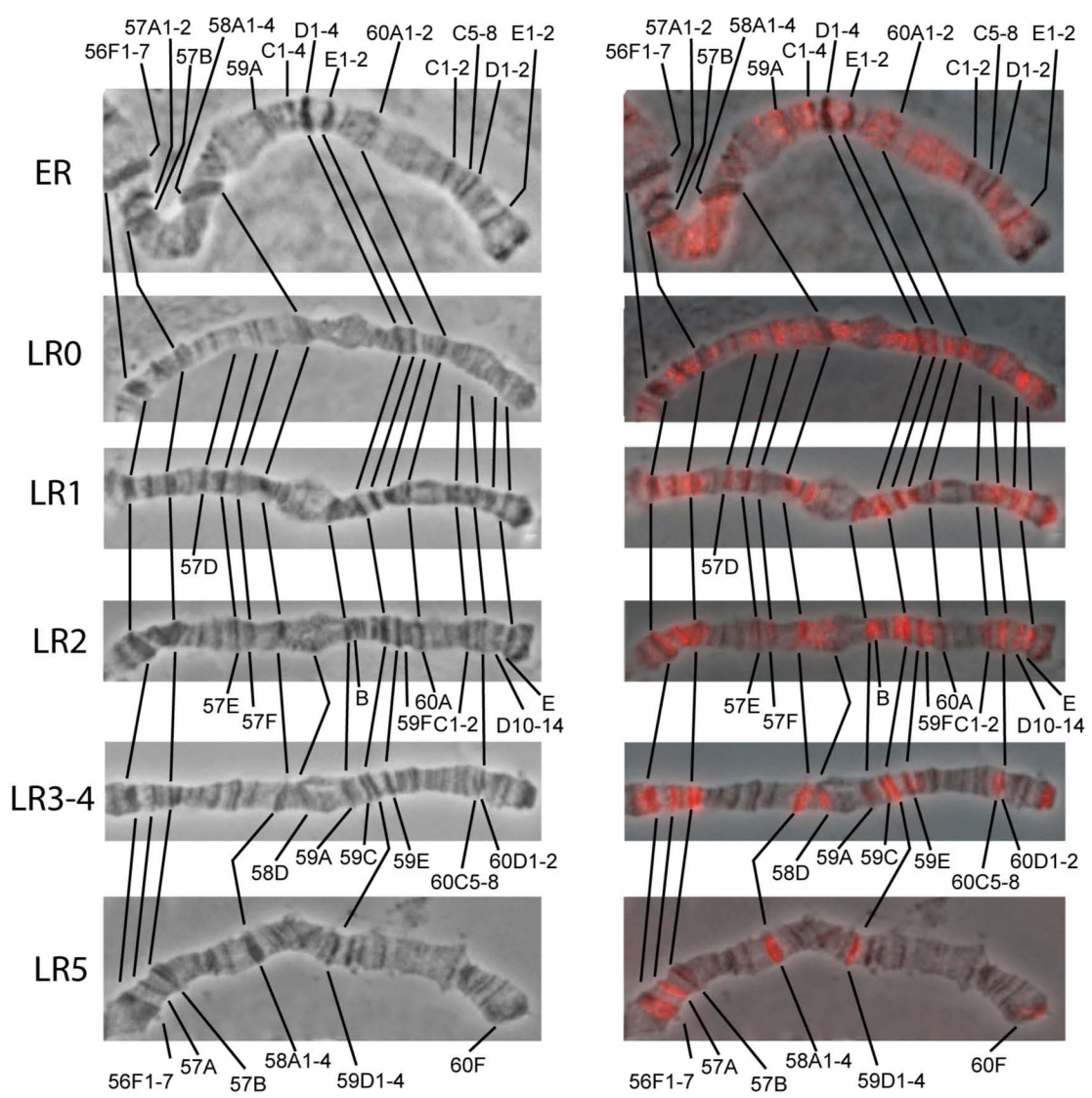

Supplement: S3 Fig — Top to bottom: six S-phase substages designated as ER (early replication) and LR0 to LR5 (late replication). Because this substaging is somewhat arbitrary and because there is variation in replication observability in separate bands at each substage, bands were assigned to substages following analysis of a large number of preparations. For this reason, the names of some substages end with more than one number. (A) Region 42-46 of chromosome 2R. (B) Regions 46-50. (C) Regions 50–56. (D) Regions 56-60. (PDF) [file pone.0195207.s003.pdf]

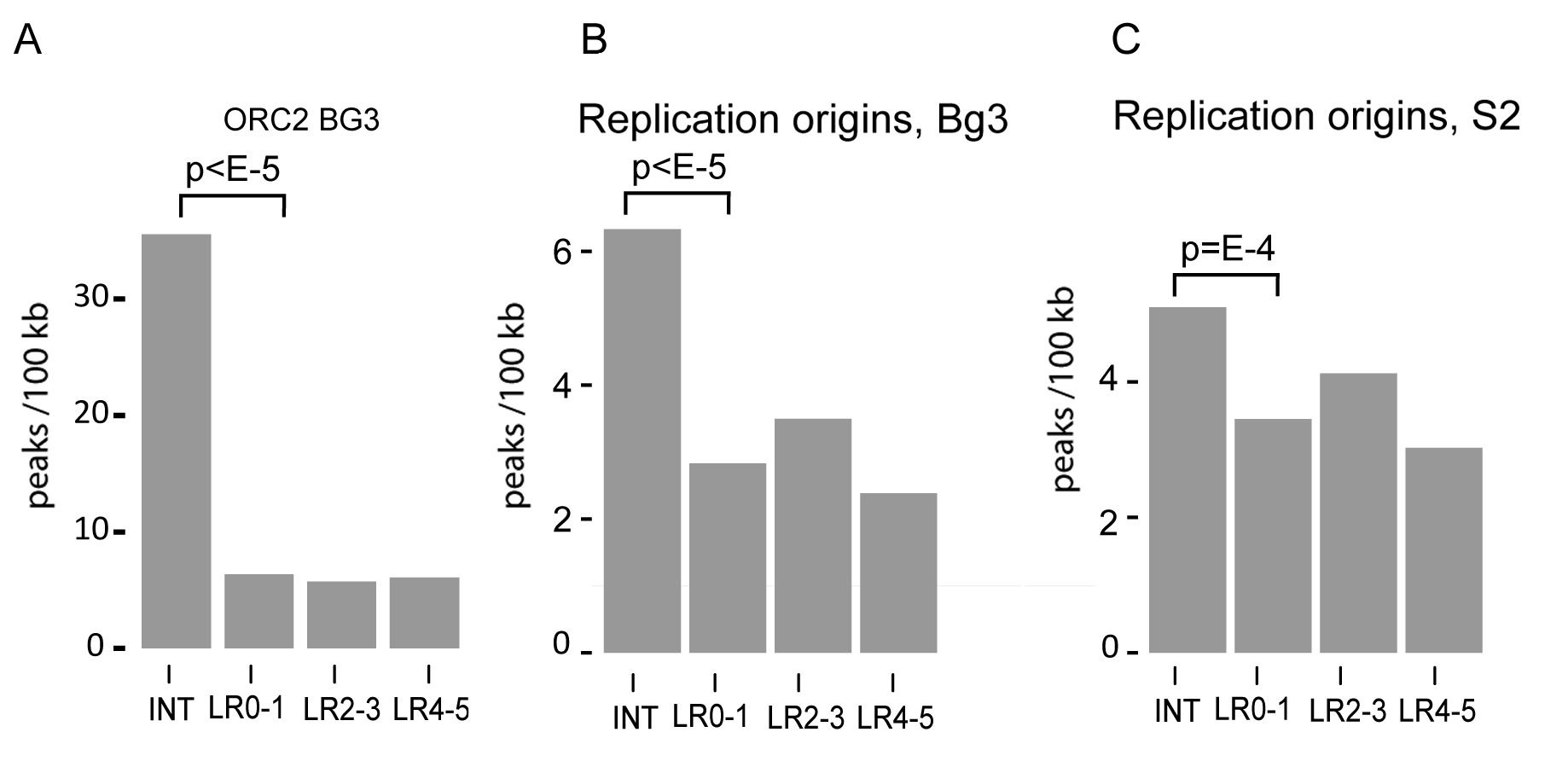

Supplement: S4 Fig — (A) Density of ORC2 peaks (peaks/100 kb) (according to [51]) in Bg3 cells. (B, C) Density of replication origins (peaks/100 kb) identified in Bg3 (B) and S2 (C) cells by next-generation sequencing of small nascent leading strands [53]. (TIF) [file pone.0195207.s004.tif]

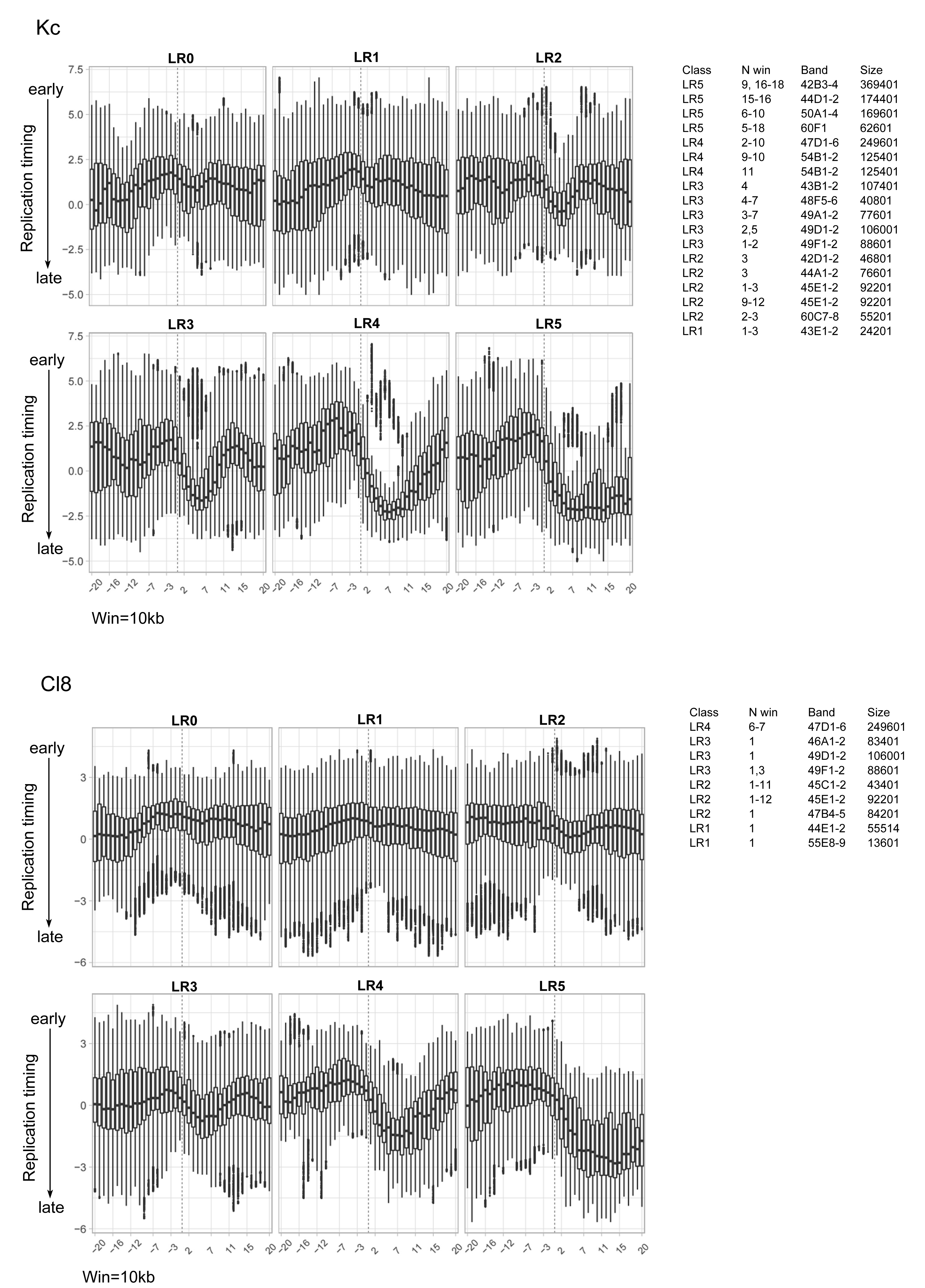

Supplement: S5 Fig — Boxplots of replication time scores in Kc (A) and Cl8 (B) cells for all sequences within 10-kb windows at the same position relative to the band boundary (shown as a red line). Right boundary data were mirrored and combined with left boundary data. Within-band regions are to the right of the border; outside regions are to the left. On the x-axis: distances from the border. On the y-axis: replication time scores (data were taken from [33]), with +6 corresponding to early replication, and −5 to late replication. On the right: all bands containing DNA sequences with replication scores corresponding to the outliers in the boxplots. For each band, the presence in a group from LR0 to LR5, the number of the windows with replication times in the outliers, and the size are indicated. To be noted most outliers correspond to the windows found at rb-band/INT borders. In Kc cells, an extended (~80 kb) outlier zone is observed in band 47D1-6 (windows 2 through 10; the total length being 250 kb). Of note, 47D1-6 is not a solid band, but a cytologically complex region, in which our interband criteria failed to identify individual bands. Extended outlier zones are observed in bands 49A1-2 (50 kb, or more than 60% of band size) and 50A1-4 (50 kb, or more than 30% of band size). In C18 cells, extended outlier zones are observed in bands 45C1-2 and 45E1-2. Here the zones span the bands and continue beyond them. In all other bands, the outlier zones are normally not longer than 20 kb each. It can be concluded that zones of changes in replication timing normally correspond to band fragments, not to entire bands. (TIF) [file pone.0195207.s005.tif]

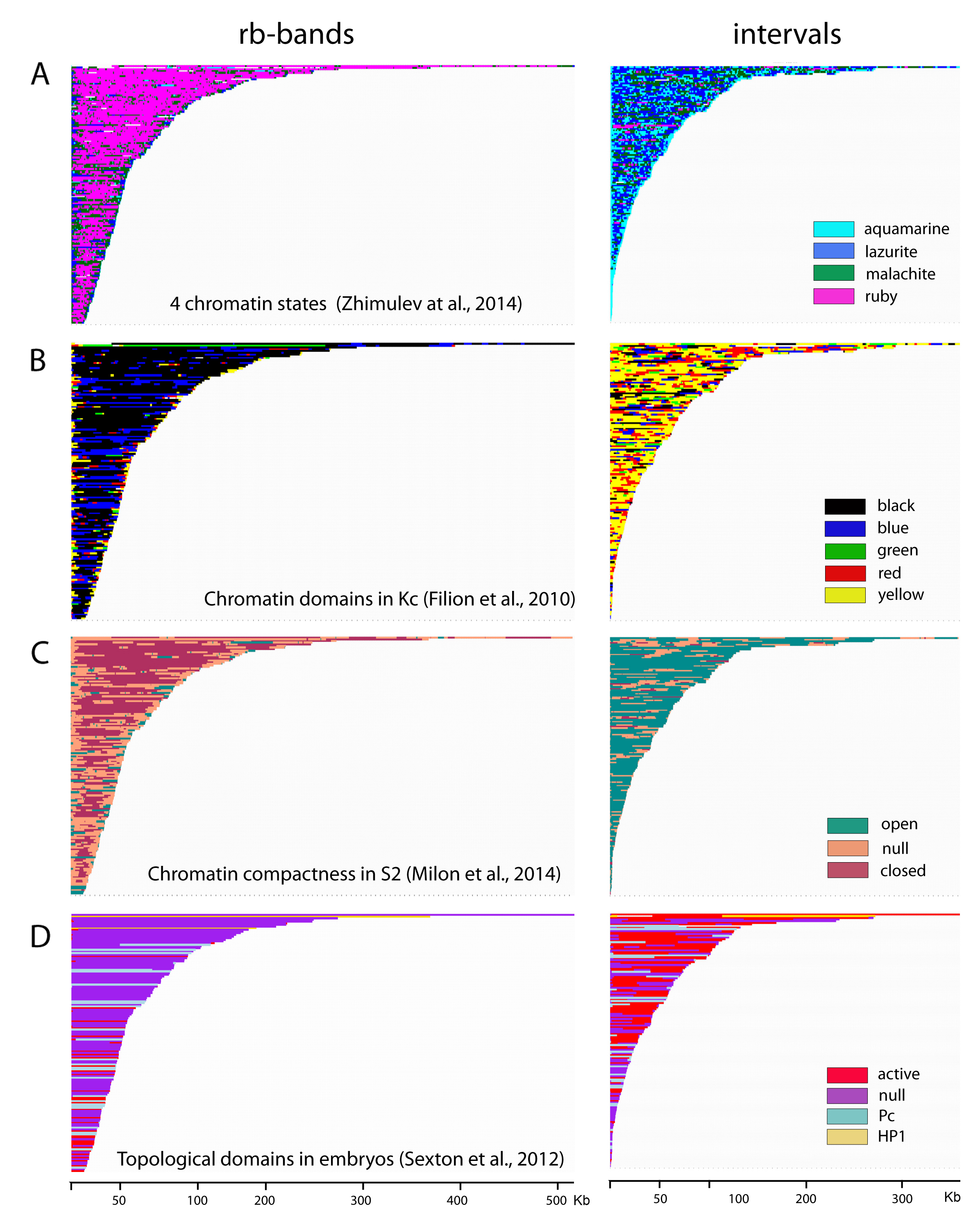

Supplement: S6 Fig — The rb-bands and INTs are presented at the same scale and are size-ranked. The scale (kb) is indicated below. (A) Distribution of four chromatin types [4]. (B) Distribution of five chromatin types in Kc cells [21]. (C) Distribution of differently compacted chromatin types in S2 cells (after [58]). (D) Distribution of four TAD types (after [30]). (TIF) [file pone.0195207.s006.tif]

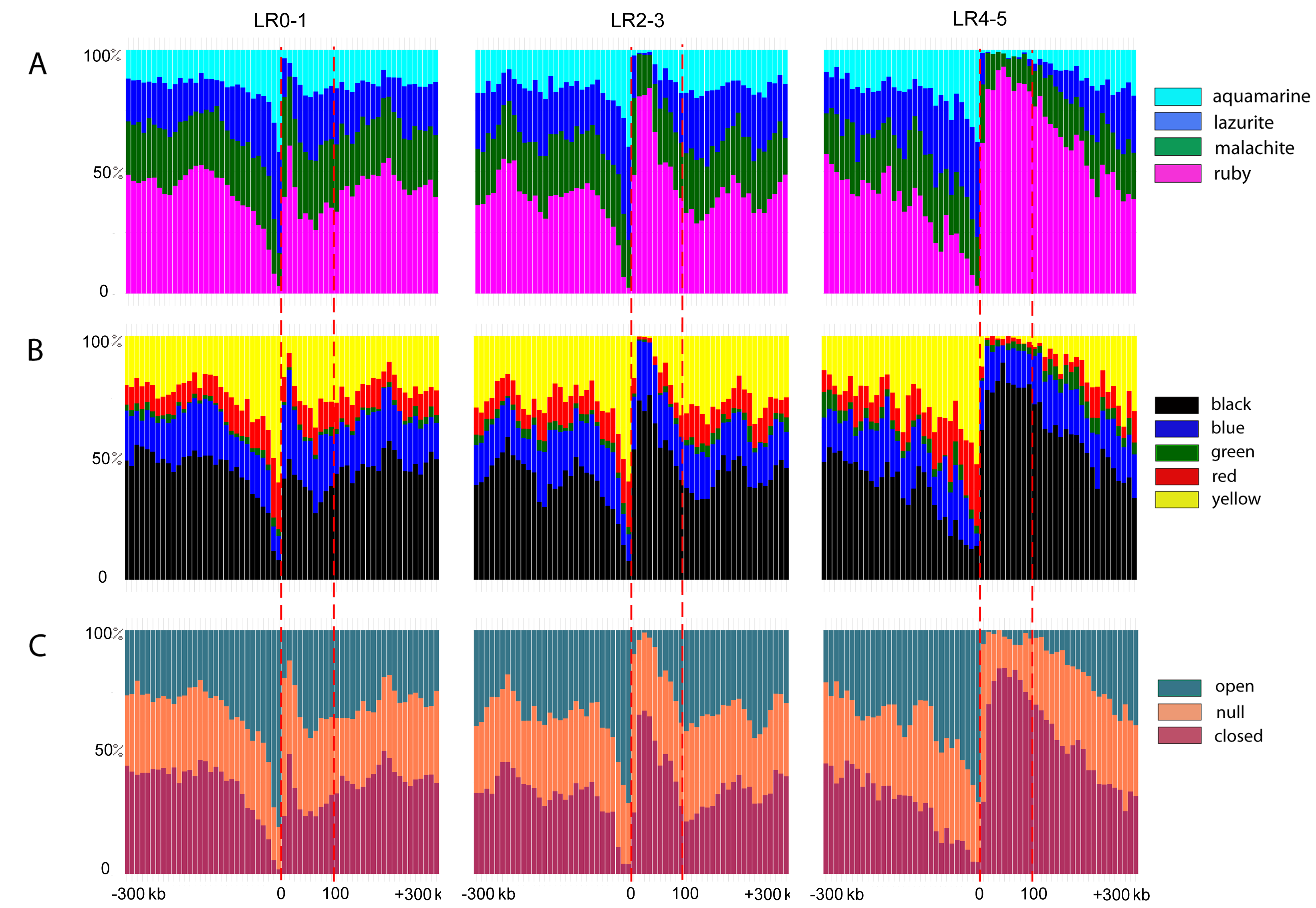

Supplement: S7 Fig — (A) An averaged distribution of four chromatin types (after [4]) at locations close to the boundaries of LR0-1, LR2-3, and LR4-5 rb-bands. (B) Averaged distribution of five chromatin types in Kc cells (after [21]) at locations close to the boundaries of LR0–1, LR2–3, and LR4–5 rb-bands. (C) An averaged distribution of differently compacted chromatin types in S2 cells [58] at locations close to the boundaries of LR0–1, LR2–3, and LR4-5 rb-bands. The band boundary is at position zero. To the right: band material; to the left: no-band material. The increment is 10 kb. Numbers of boundaries analyzed: for LR0-1/INT n = 113, for LR2-3/INT n = 76, and for LR4-5/INT n = 36. (TIF) [file pone.0195207.s007.tif]

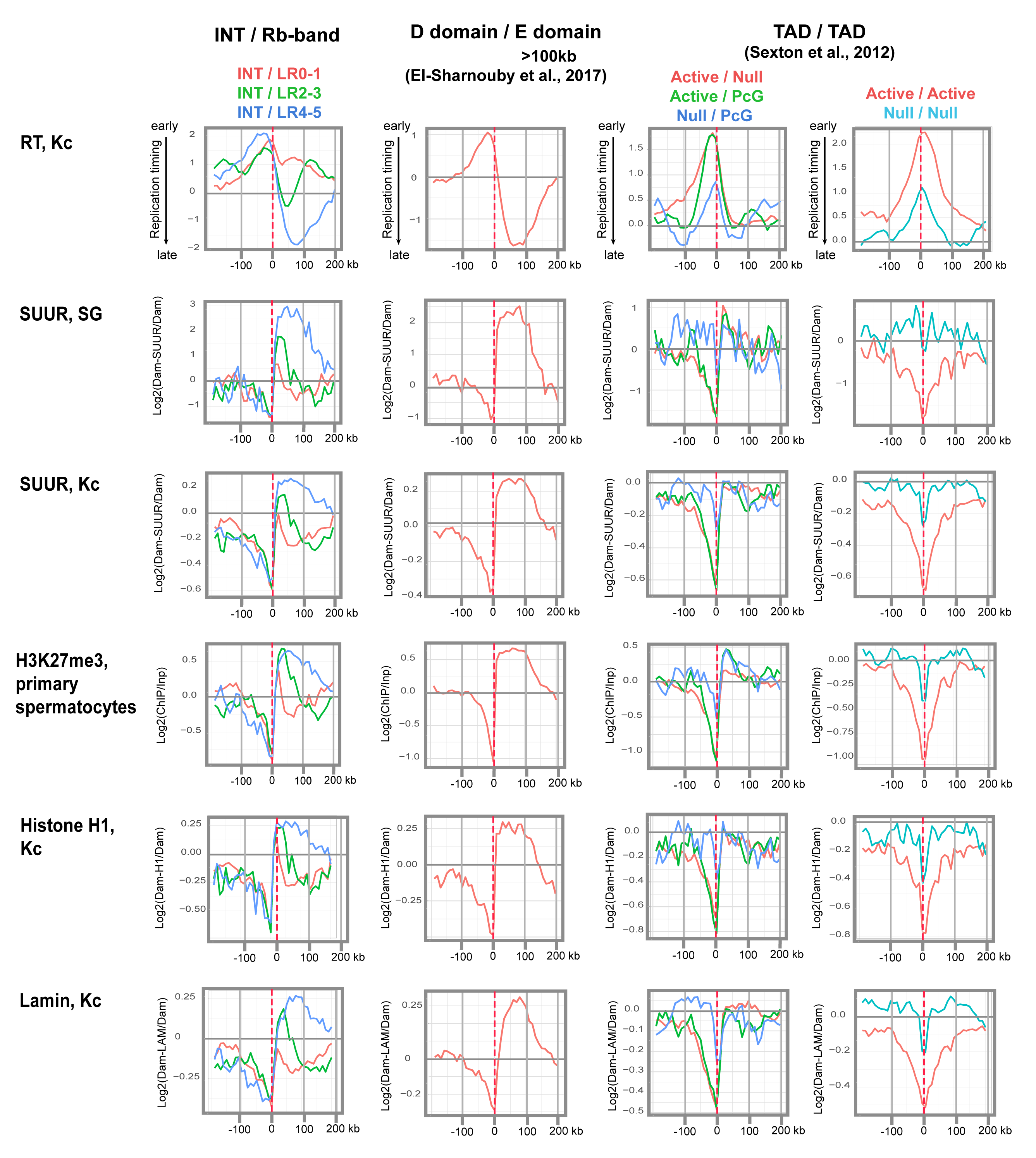

Supplement: S8 Fig — Averaged profiles of the distribution of scores for repressed chromatin markers close to the boundaries between rb-bands and INTs, D- and E-domains (E domains less than 100 kb were removed), TADs of different types (null/active, Pc/active, null/Pc), and TADs of the same type (null/null, active/active). Right-boundary data were mirrored and combined with left boundary data. Markers: SUUR in salivary glands (data were taken from [59]), SUUR in Kc cells data were taken from [21]), LAMIN in Kc cells (data were taken from [60]), histone H1 in Kc cells (data were taken from [21], and H3K27Me3 in primary spermatocytes (after [31]). Boundaries that were closer than 10 kb apart were removed from the analysis. Numbers of boundaries analyzed: for LR0-1/INT n = 113, for LR2-3/INT n = 76, for LR4-5/INT n = 36. For D/E > 100 kb boundaries n = 832. For TAD/TAD boundaries: Active/Null n = 246, Active/Pg n = 62, Null/Pg n = 49, Active/Active n = 174, Null/Null n = 189. (TIF) [file pone.0195207.s008.tif]

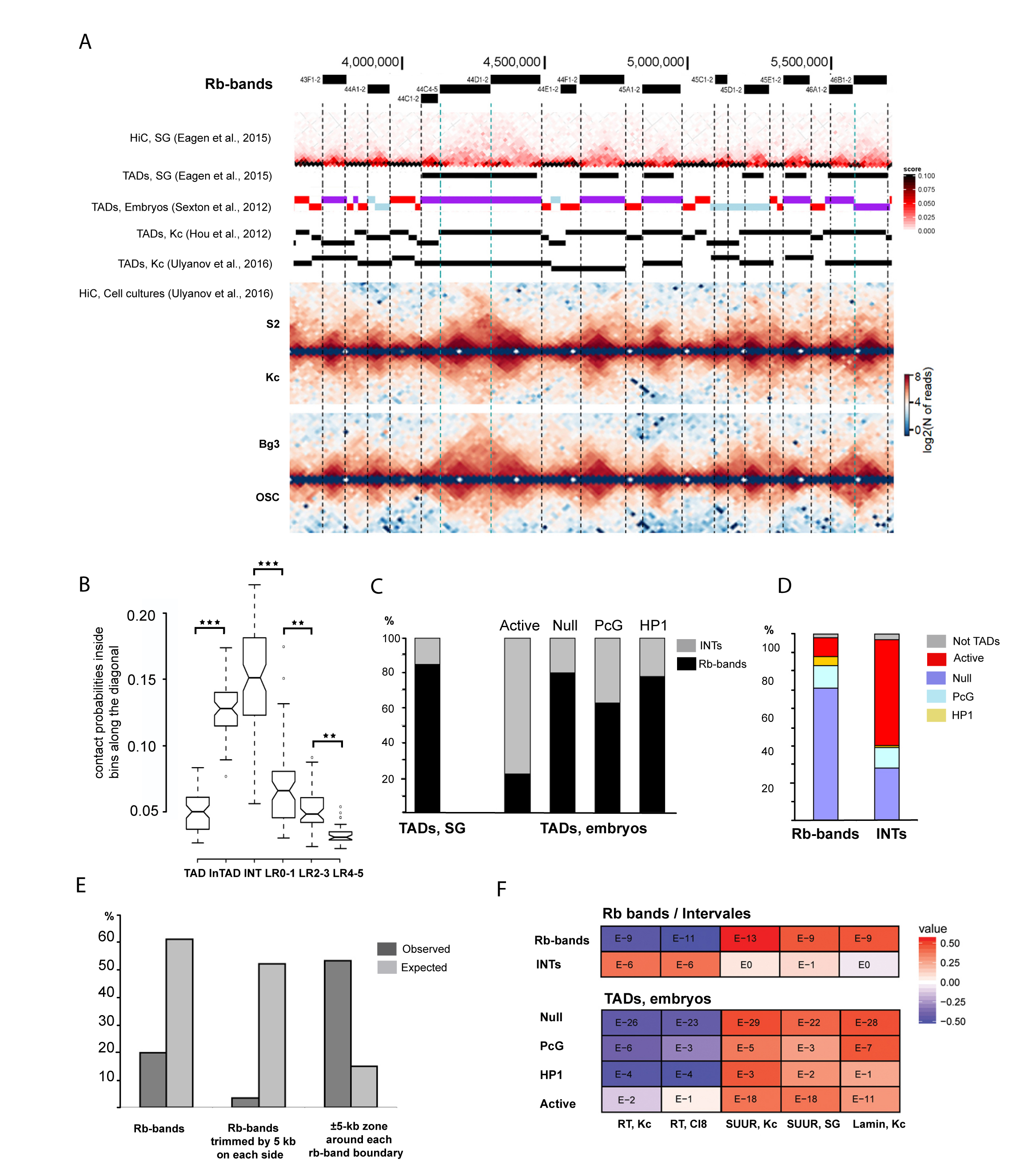

Supplement: S9 Fig — (A) Hi-C data from different sources on the region 45F-46B of chromosome arm 2R (approximately 2 Mb). Top to bottom: rb-bands; a normalized Hi-C interaction map (15-kb bins) from salivary gland cells [26]; TADs in salivary gland cells [26]; TADs in embryonic cells according to [30]: null type (purple), PcG type (blue), active type (red); TADs in Kc167 cultured cells [29]; Hi-C interaction maps (20-kb bins) of the four cell lines [27]. (B) Chromatin packaging in all rb-bands corresponds to chromatin packaging in TADs. Boxplots of contact probabilities inside bins along the diagonal on the normalized Hi-C interaction map published by Eagen et al. [26] for salivary gland cells. TADs are significantly different from interTADs (inTAD in the figure; ***p < E-21); all rb-bands are significantly different from INTs (***p < E-16; **p < E-3; Mann–Whitney U test). (C) The ratio of the overall lengths of genomic intervals corresponding to LR0–LR5 bands (black) and INTs (grey) in TADs of different types. In all inactive TAD types, the prevalent chromatin is the one corresponding to bands. (D) The proportion ratio of chromatin types corresponding to TADs of different types according to [30], in LR0-LR5 bands and intervals between them. (E) Rb-bands are depleted of TAD boundaries in embryos. The observed and the expected number (assuming a normal distribution along the chromosome) of TAD boundaries (after [30]) within rb-bands and at their boundaries. (F) Coefficients of correlation (with p values as indicated) between fragment size and such measures as replication timing in Kc and Cl8 cells, SUUR binding in Kc and salivary gland cells, and Lamin binding in Kc cells. (TIF) [file pone.0195207.s009.tif]

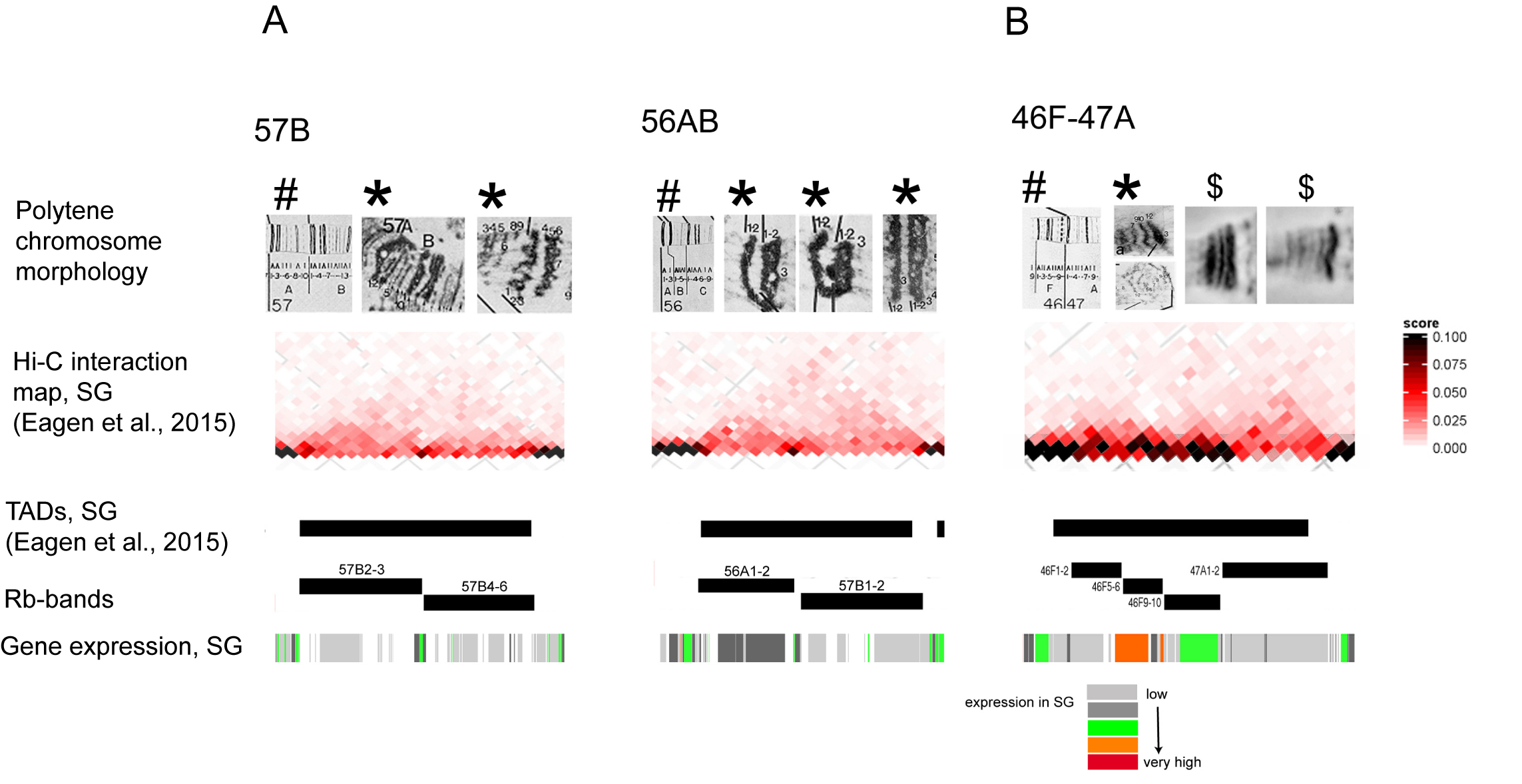

Supplement: S10 Fig — Top to bottom: polytene chromosome morphology (#according to Bridges, 1939 [47]; *an electron micrograph according to [45]; $aceto-orcein staining, phase-contrast); the heatmap of Hi-C data on salivary glands [26]; TADs in salivary glands [26]; rb-bands; gene expression levels (according to modENCODE [43]). (A) Each pair of closely spaced bands, 57B2-3/57B4-6 and 56A1-2/56B1-2, forms a single TAD. The internal interband manifests a very special feature on the heatmap: within the triangles with nearly evenly distributed interactions, the interband bin shows strong interactions between internal sequences and weak interactions between external. (B) In region 46F, polytene chromosome morphology is variable: what is seen to the left of the large and always prominent band 47A1-2 is sometimes a group of distinct black bands and sometimes a series of decompacted grey bands. This variability is probably associated with the activity of the Hr3 gene (the orange rectangle on the expression map), which, according to modENCODE, starts to be highly expressed shortly before the prepupal stage. The heatmap interval corresponding to this variable region showed alternations of bins with different properties and looked contrasting against extended regions with relatively uniform patterns of interactions. (TIF) [file pone.0195207.s010.tif]
