# Supplementary material for: Similarity in replication timing between polytene and diploid cells is associated with the organization of the Drosophila genome
Source: PLoS One. 2018 Apr 16;13(4):e0195207. doi: 10.1371/journal.pone.0195207 (PMC5902040; doi:10.1371/journal.pone.0195207)
Supplement: S1 Text — (PDF) [file pone.0195207.s015.pdf]

Supplementary Text 1

All figures and references in Supplementary Text are enumerated independently from the main text.

Mapping strategy, as exemplified by the bands 44F1-2 and 45A1-2

1. Morphology analysis

The bands labeled as 44F1-2 and 45A1-2 on the Bridges' detailed map (Bridges, Bridges, 1939) (red arrows) are clearly visible at both the light microscopy and electron microscopy levels (EM map from Saura, 1986, available in FlyBase: https://wiki.flybase.org/wiki/FlyBase:Maps) and appear as compact black bands with clear-cut boundaries (Fig 1).

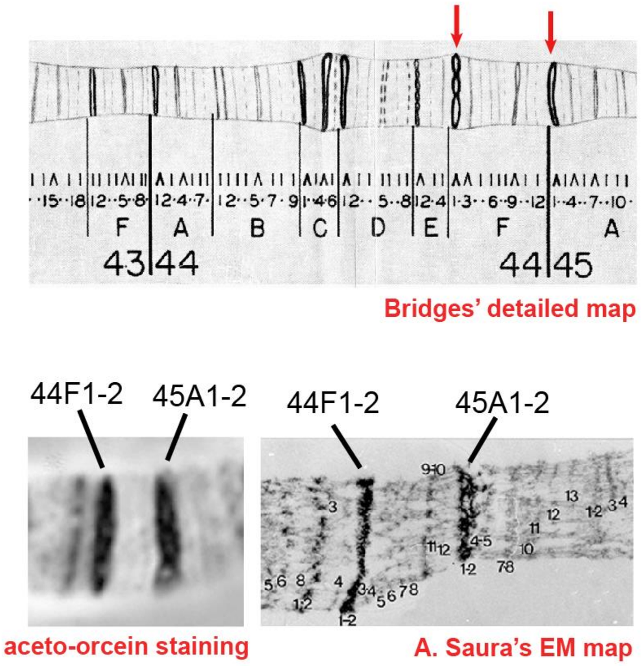

Figure 1

2. Identification of the appropriate area in the UCSC genome browser using the "Chromosome Band track" (chromosome bands annotated by FlyBase). Approximate localization of ruby-containing polytene chromosome bands on the genomic map by selecting the zones containing ruby chromatin and flanked by aquamarine chromatin (according to the 4 state model by Zhimulev et al., 2014).

There are three zones of enrichment for ruby chromatin in the region of interest (Fig 2, magenta color). The assumption is that they correspond to three bands: 44E1-2, 44F1-2, 45A1-2. Assuming that all bands are flanked by interbands, and that all interbands fall into the aquamarine chromatin, the putative boundary position is specified as the first nucleotide next to the aquamarine region. Thus, a set of predicted rb-bands is formed (Fig 2).

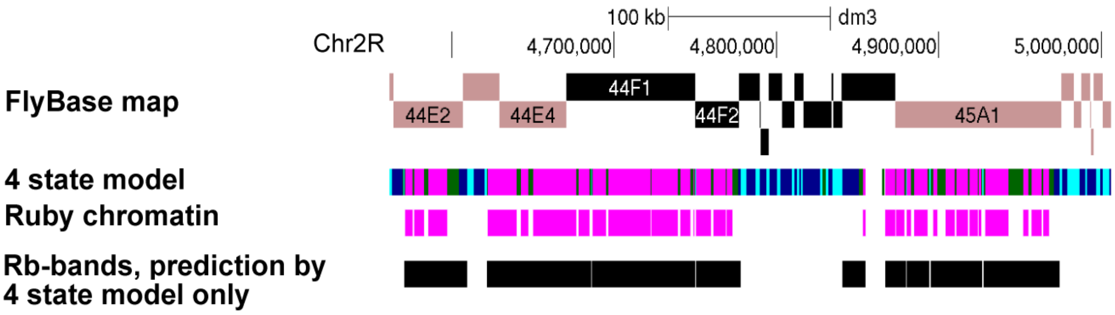

Figure 2

3. Correction of the band boundary prediction based on the distribution of CHRIZ protein and TSS-enriched chromatin in the chromosomes from *Drosophila* cell lines.

It was shown that not all aquamarine fragments correspond to interbands (Zhimulev et al., 2014, Khoroshko et al., 2016, Boldyreva et al., 2017). Based on the published data, we presumed that all aquamarine fragments that contain CHRIZ binding peaks in the four cell cultures and overlap with type 1 chromatin (active promoters, shown red in the figure) under the 9-state model (Kharchenko et al., 2011) in both Bg3 and S2 cells have a high probability to be interbands. The corrected band positions are presented in the Fig 3.

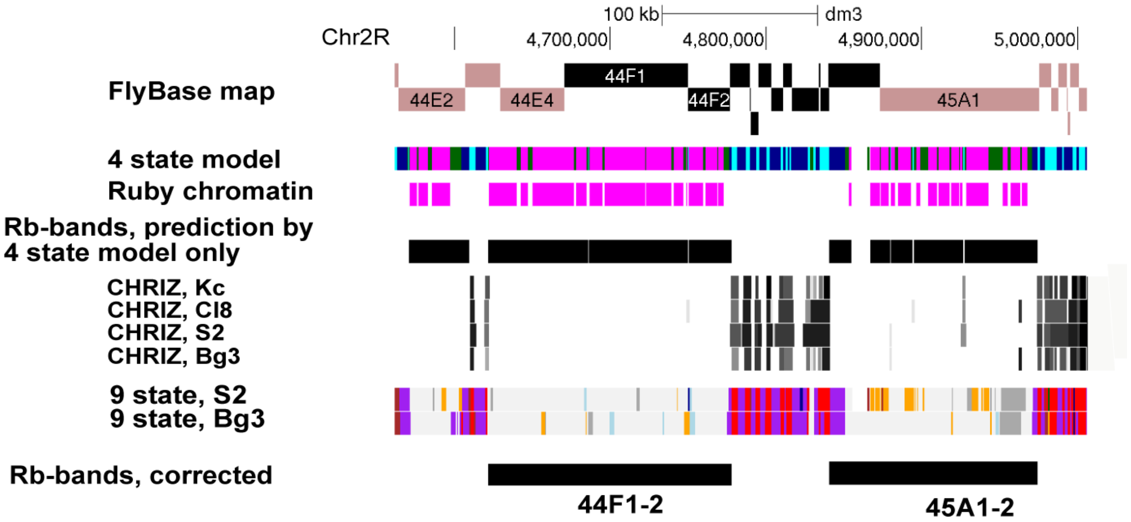

Figure 3

In the case described, this correction perfectly matches the morphology of polytene chromosomes: 44F1-2 and 45A1-2 are clear solid bands, with no detectable interbands even at the EM level (Fig 1).

5. Refinement of the nomenclature and boundaries of the predicted bands using the "experimental cytology" data from FlyBase.

FlyBase-referenced genes having experimental mapping data were extracted (see Materials and Methods). Within the region of interest, there are 21 such genes. These genes were plotted on the map below, with the mapping data summarized in the Table 1.

.

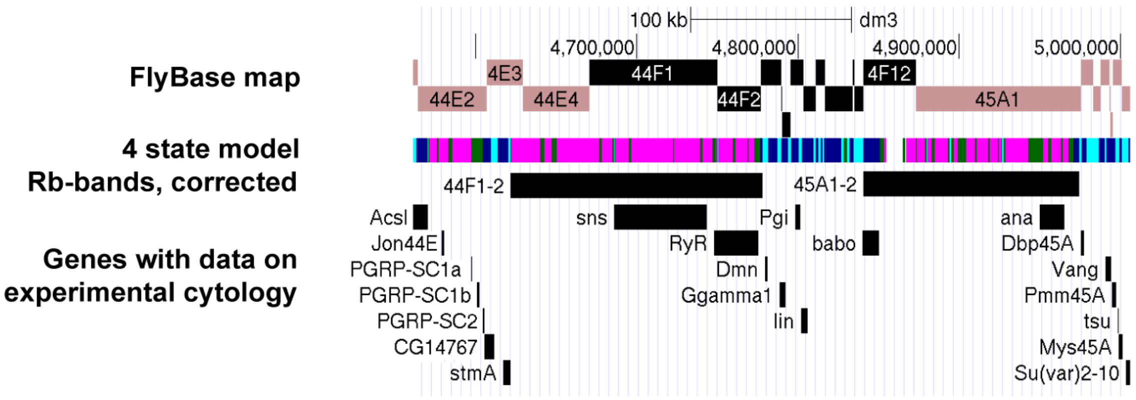

Figure 4

Acsl	44D3-44D6; 44F1-44F2 (ISH*); 44E1--2 (ISH); 44E-44E (ISH); 24D5-24D6; 44D6—8; 44D8--E1
Jon44E	44E-44E (ISH)
PGRP-C1a	44E-44E
PGRP-C1b	44E-44E
PGRP-SC2	44E-44E
CG14767	44D8-44D8
stmA	44D1-44D2; 4E3-44E4
sns	44F-44F; 43-49
RyR	44F-44F; 44F1-44F2 (ISH); 44F-44F (ISH)
Dmn	44F3-44F4 (ISH); 44F2-44F6; 44F3--4
Ggamma1	44F3-44F4 (ISH); 44F3-44F4; 44C-44D (ISH)
Pgi	44F9-45A2 (ISH)
lin	44F-44F
babo	45A1-45A2; 44F11-44F12 (ISH); 44F11-44F12; 45A1—2; 45A1-45A2 (ISH)
ana	45A-45B (ISH)
Dbp45A	45A-45A; 45A-45A (ISH)
Vang	45A7-45A10; 45A-45B (ISH)
Pmm45A	45A-45A
tsu	45A-45A; 45A4-45A4 (ISH)
Mys45A	45A-45A
Su(var)2-10	45A4-45A8; 45A4-45A8 (ISH)

* ISH – determined by in situ hybridization.

Table 1

Data from different papers differ significantly in the mapping accuracy for the same gene. Therefore, the above data should be used with caution. Whenever necessary, we

followed the direct FlyBase links to verify mapping details. Unfortunately, the overwhelming majority of articles do not feature the images showing the results of mapping. Nevertheless, in aggregate, these data were very useful.

For the genes found to the left of the putative 44F1-2 band, the mapping results are referred to as 44D-E. Note the *stmA* gene: its promoter lies in the interband next to 44F1-2, and the body is directed out of the band, so was mapped to the left of 44F1-2.

Both *sns* and *RyR* genes that we believe are found within the 44F1-2 band are annotated as mapping to 44F1-2 or 44F.

According to our prediction, the *Dmn* gene lies in the interband flanking the band 44F1-2 to the right. Most of the mapping data available lie to the right of 44F1-2 (44F3-4).

Data on the experimental mapping for the genes *Ggamma1, Pgi* and *lin* confirm our prediction that these genes lie between the 44F1-2 and 45A1-2 bands.

The gene *babo* has a promoter in the aquamarine chromatin delimiting the putative 44A1-2 band, whereas the gene body lies in the band. Some references localize this gene to the left of the band 45A1-2 (as 44A11-12), other reports place it into the band 45A1-2 itself. FlyBase references the paper (Spradling et al., 1999) focusing on p-element insertion mapping, where this gene maps to the region 44F11-12. In particular, the insertion *P{lacW}babo*^{k16912} found in the gene promoter was mapped to 44A11-12. This corresponds to our prediction that promoter of the *babo* gene lies in the interband adjacent to the 45A1-2 band.

For the *ana* gene predicted inside the 45A1-2 band and the *Dbp45A*, *Pmm45A*, *Mys45* genes predicted to be found to the right, the mapping region is very broad (45A).Yet, *Vang, tsu,* and *Su(var)2-10* genes are mapped to the right of the 45A1-2 band.

Thus, the “experimental cytology” data agree well with the assignment of the two predicted bands to 44F1-2 and 45A1-2 and provide additional information about the real position of the band boundaries in polytene chromosomes.

6. Refinement of the band boundary prediction based on the comparison with data on gene expression and protein localization obtained for polytene chromosomes.

To visualize the position of genes that are highly expressed in salivary glands, a custom color-coded gene expression track was created in the UCSC browser. In the case of 44F1-2 and 45A1-2 bands, there are no genes within the bands that are expressed at high or even moderate levels, with the exception of one gene on the left border of the 45A1-2 (Fig

5). It can be assumed that in this case tissue-specific expression should not influence the prediction of the band boundaries.

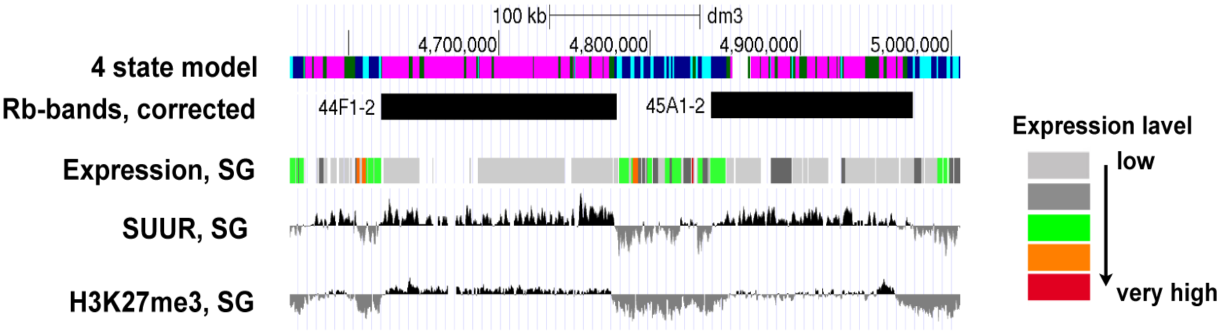

Figure 5

In the chapter “Rb-bands are significantly enriched for repressive chromatin types and display a sharp change in chromatin protein density at their peripheries“ we show that rb- bands borders coincide with sharp changes on the profiles of the SUUR protein as well as of other markers of repressive chromatin. Very recently, the distribution profiles for SUUR and H3K27me3 in the salivary glands have been published (Posukh et al., 2017). On the Fig 5 we compare these profiles with our mapping of 44F1-2 and 45A1-2. Both bands correspond to the zones where both SUUR and H3K27me3 are enriched, with pronounced drops in the profiles exactly matching the positions of the predicted boundaries. Fig 6 shows a close-up of the right border of the 44F1-2. The transition through the zero point on the SUUR profile in this case very accurately corresponds to the predicted boundary of the band. The transition through the zero point on the profile of H3K27me3 is shifted by 2 kb to the left. We believe that the boundary mapping accuracy in this case is about 2 kb.

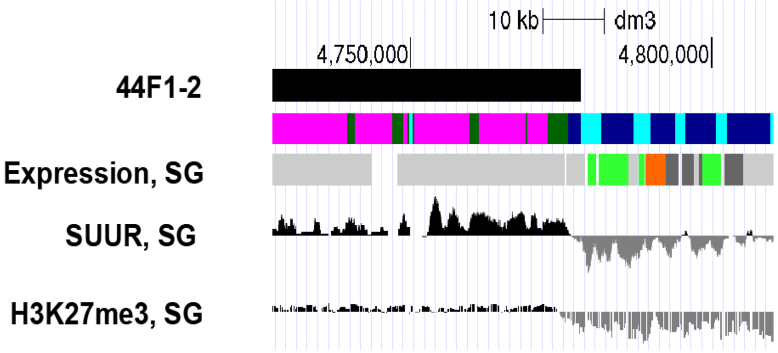

Figure 6

The bands 44F1-2 and 45A1-2 represent an example of the situation when the morphology is clear, the nomenclature is understandable, the data obtained on polytene

chromosomes of the salivary glands and the data obtained on cell cultures do not contradict each other.

Examples of discordant cases

For several bands the data obtained from cell cultures have come into conflict with the data obtained on salivary glands. There are examples where data obtained on cell cultures predict a rb-band, but no such band is observed in polytene chromosomes. For example, we can see a group of thin bands 47F8-14 instead of a single black band (Fig 7, red arrow). This region encompasses a gene *Dril*, which is characterized by a very high level of expression in salivary gland. A track that visualizes genes with a high level of expression makes it easy to predict bands where expression can affect mapping.

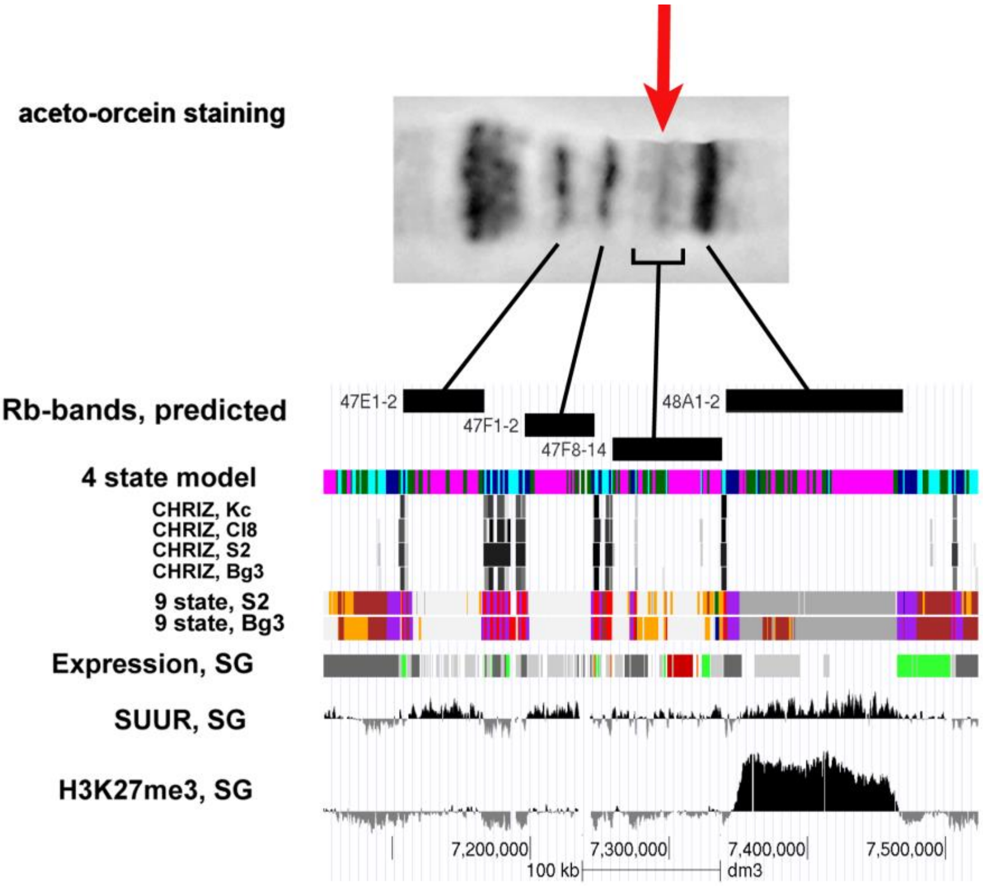

Figure 7

Very high transcription levels may not necessarily affect the results of mapping. For example, in 46A1-2, modEncode predicts a high level of expression of the *Mmp2* gene covering almost the entire band. According to the EM analysis, when transcription is activated, a "dark" puff is formed from this band, that is, the band becomes morphologically slightly more decompacted (Semeshin et al., 2001).

There are a handful of cases that are particularly difficult to map because of the atypical morphology of the polytene chromosome band or a group of bands. For example, 47D1-6 and 49E5-7-F1-2 in 2R. At the edges, they are flanked by morphologically distinct and well-predicted interbands. No interbands are visible inside, yet the chromatin properties, in particular, the distribution of SUUR and H3K27me3 are very heterogeneous. In the case of 47D1-6 (Fig 8, red arrows), the left half is compact, and it is the left half that is enriched with SUUR and H3K27me3 (Fig 8).

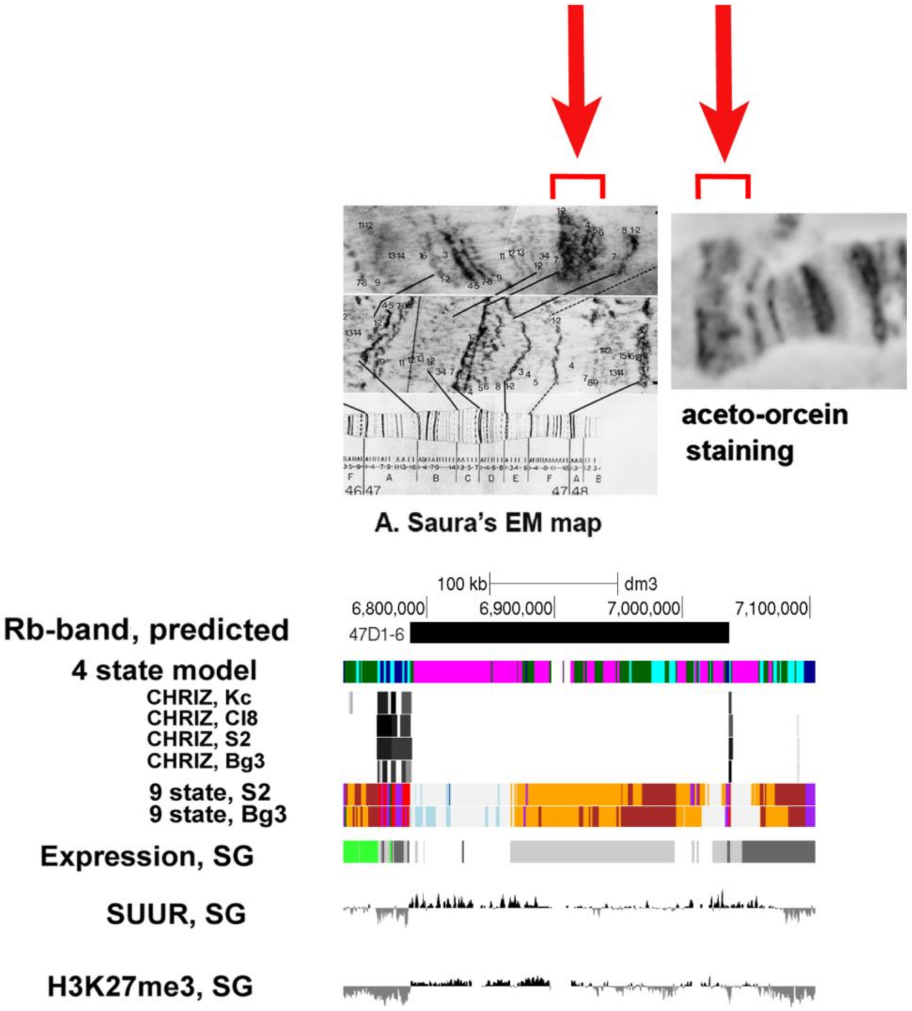

Figure 8

In the case of 49F1-2, the compact material of the band appears not to be bordered by typical interband to the left, but rather by the chromatin with other properties (Fig 9). These cases are rare and require a separate study.

All these examples indicate that careful case-by-case analysis is needed to obtain a high resolution mapping of each band. Fortunately, the data presently available provide an opportunity to achieve good mapping results.

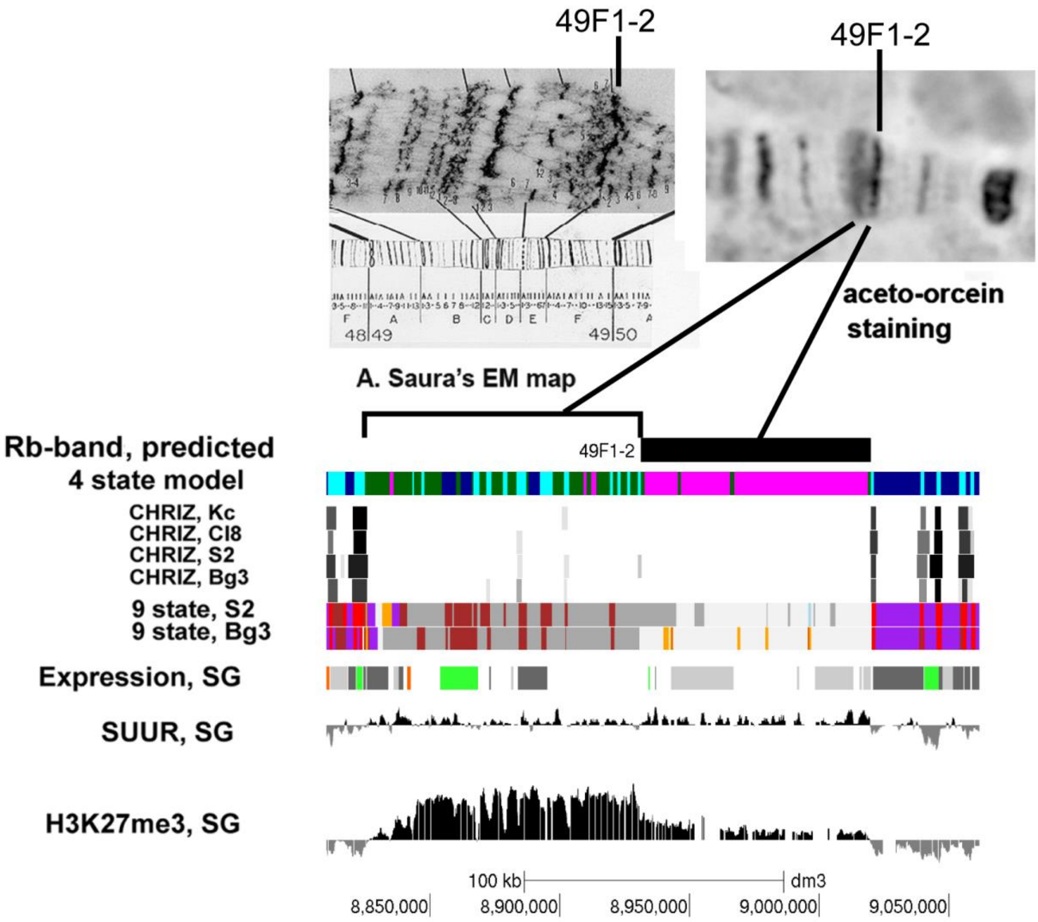

Figure 9

References

Boldyreva LV, Goncharov FP, Demakova OV, Zykova TY, Levitsky VG, et al. (2017) Protein and Genetic Composition of Four Chromatin Types in Drosophila melanogaster Cell Lines Curr Genomics 18: 214-226.

Bridges CB, Bridges PN (1939) Reference map of the salivary gland 2R-chromosome of Drosophila melanogaster. J Hered 30: 475-476.

Kharchenko PV, Alekseyenko AA, Schwartz YB, Minoda A, Riddle NC, et al. (2011) Comprehensive analysis of the chromatin landscape in Drosophila melanogaster. Nature 471: 480-485.

Khoroshko VA, Levitsky VG, Zykova TY, Antonenko OV, Belyaeva ES, et al. (2016) Chromatin Heterogeneity and Distribution of Regulatory Elements in the Late-Replicating Intercalary Heterochromatin Domains of Drosophila melanogaster Chromosomes. PLoS One 11: e0157147.

Posukh OV, Maksimov DA, Laktionov PP, Koryakov DE, Belyakin SN. (2017) Functional dissection of Drosophila melanogaster SUUR protein influence on H3K27me3 profile. Epigenetics Chromatin. 2017 Dec 1;10(1):56. doi: 10.1186/s13072-017-0163-z.

Saura A (1986) Electron microscopic mapping of the second polytene chromosome of Drosophila melanogaster University of Helsinki. 58 p.

Saura AO, Heino TI, Sorsa V (1991) Electron micrograph maps of divisions 51 through 60 of thin sectioned polytene 2R chromosome of Drosophila melanogaster. Hereditas 114: 15-34.

Semeshin VF, Shloma VV, Zhimulev IF. Formation and morphology of dark puffs in Drosophila melanogaster polytene chromosomes.Hereditas. 2001;134(1):15-22.

Spradling AC, Stern D, Beaton A, Rhem EJ, Laverty T, et al. (1999) The Berkeley Drosophila Genome Project gene disruption project: Single P-element insertions mutating 25% of vital Drosophila genes. Genetics 153: 135-177.

Zhimulev IF, Zykova TY, Goncharov FP, Khoroshko VA, Demakova OV, et al. (2014) Genetic organization of interphase chromosome bands and interbands in Drosophila melanogaster. PLoS One 9: e101631.
